# The influence of molecular markers and methods on inferring the phylogenetic relationships between the representatives of the *Arini* (parrots, Psittaciformes), determined on the basis of their complete mitochondrial genomes

**DOI:** 10.1186/s12862-017-1012-1

**Published:** 2017-07-14

**Authors:** Adam Dawid Urantowka, Aleksandra Kroczak, Paweł Mackiewicz

**Affiliations:** 1 Department of Genetics, Wroclaw University of Environmental and Life Sciences, ul. Kożuchowska7, 51-631, Wroclaw, Poland; 2 Department of Genomics, Faculty of Biotechnology, University of Wrocław, ul. Fryderyka Joliot-Curie 14a, 50-383 Wrocław, Poland

**Keywords:** *Arini*, Molecular markers, Parrots, Phylogenetic methods, Phylogenetic tree, Psittaciformes

## Abstract

**Background:**

Conures are a morphologically diverse group of Neotropical parrots classified as members of the tribe *Arini*, which has recently been subjected to a taxonomic revision. The previously broadly defined *Aratinga* genus of this tribe has been split into the ‘true’ *Aratinga* and three additional genera, *Eupsittula*, *Psittacara* and *Thectocercus*. Popular markers used in the reconstruction of the parrots’ phylogenies derive from mitochondrial DNA. However, current phylogenetic analyses seem to indicate conflicting relationships between *Aratinga* and other conures, and also among other *Arini* members. Therefore, it is not clear if the mtDNA phylogenies can reliably define the species tree. The inconsistencies may result from the variable evolution rate of the markers used or their weak phylogenetic signal. To resolve these controversies and to assess to what extent the phylogenetic relationships in the tribe *Arini* can be inferred from mitochondrial genomes, we compared representative *Arini* mitogenomes as well as examined the usefulness of the individual mitochondrial markers and the efficiency of various phylogenetic methods.

**Results:**

Single molecular markers produced inconsistent tree topologies, while different methods offered various topologies even for the same marker. A significant disagreement in these tree topologies occurred for *cytb*, *nd2* and *nd6* genes, which are commonly used in parrot phylogenies. The strongest phylogenetic signal was found in the control region and RNA genes. However, these markers cannot be used alone in inferring *Arini* phylogenies because they do not provide fully resolved trees. The most reliable phylogeny of the parrots under study is obtained only on the concatenated set of all mitochondrial markers. The analyses established significantly resolved relationships within the former *Aratinga* representatives and the main genera of the tribe *Arini*. Such mtDNA phylogeny can be in agreement with the species tree, owing to its match with synapomorphic features in plumage colouration.

**Conclusions:**

Phylogenetic relationships inferred from single mitochondrial markers can be incorrect and contradictory. Therefore, such phylogenies should be considered with caution. Reliable results can be produced by concatenated sets of all or at least the majority of mitochondrial genes and the control region. The results advance a new view on the relationships among the main genera of *Arini* and resolve the inconsistencies between the taxa that were previously classified as the broadly defined genus *Aratinga*. Although gene and species trees do not always have to be consistent, the mtDNA phylogenies for *Arini* can reflect the species tree.

**Electronic supplementary material:**

The online version of this article (doi:10.1186/s12862-017-1012-1) contains supplementary material, which is available to authorized users.

## Background

The most species-rich group of the New World parrots is the subfamily *Arinae* [1, 2]. In the present taxonomy, it includes 164 species classified into 35 extant genera and one extinct genus *Conuropsis* [3]. A remarkable feature of this group is the extraordinarily high diversity in morpho-behavioural characters across its genera and even species [4]. Understandably, the exact number of genera and species belonging to *Arinae* still remains questionable and controversial. The current formal separation of some *Arinae* taxa is often based on quite intuitive and subjective morphological differences. Molecular analyses do not provide conclusive results and the phylogenetic position of many *Arinae* taxa varies depending on the published molecular datasets [5, 6]. At present, the subfamily *Arinae* is divided into the following tribes: [3] *Amoropsittacini*, *Androglossini*, *Arini* and *Forpini*. Among them, the *Arini* is represented by the largest number of genera. Of the 19 currently recognized genera, nine (*Aratinga*, *Enicognathus*, *Eupsittula*, *Guaruba*, *Leptosittaca*, *Ognorhynchus*, *Psittacara*, *Pyrrhura* and *Thectocercus*) form a morphologically diverse group called conures [7].

From the taxonomical point of view, the genus *Aratinga* is arguably the most controversial albeit noteworthy. Until now, the genus has been defined in different ways. One of the researchers identified 23 species within the genus [2]. Notwithstanding, Ribas and Miyaki [8]as well as Tavares et al. [9] called into question the monophyly of the genus. Silveira et al. [10] provided also some molecular details showing that the genus consists of three separate lineages, which were confirmed by further studies based on much broader taxon sampling [6, 11, 12]. Finally, Urantowka et al. [6] supplied the first molecular data for *Aratinga acuticaudata* and found that the species is more closely related to the representatives of three monotypic genera (*Diopsittaca*, *Guaruba* and *Leptosittaca*) than to any other member of the genus *Aratinga*. *Aratinga acuticaudata* was formerly included by Silveira et al. [10] in one of three separated clades of *Aratinga*, drawing on only some morphological features. The delimitation of the clade consisting of *Aratinga acuticaudata*, two other conures (*Guaruba*, *Leptosittaca*) and the smallest macaw (*Diopsittaca nobilis*) resulted in reviving the monotypic genus *Thectocercus* for the former *Aratinga acuticaudata*. By virtue of the molecular revision of the broadly defined genus *Aratinga*, two additional genera, *Eupsittula* and *Psittacara*, were elevated for two of three previously recognized *Aratinga* lineages, whereas the ‘true’ *Aratinga* genus was established as the third lineage [7].

The currently recognized sensu stricto genus *Aratinga* comprises six South American species: *auricapillus*, *jandaya*, *maculata*, *nenday*, *solstitialis* and *weddellii*. *Aratinga solstitialis* and *Aratinga nenday* are two species which are predominantly used as representatives of this genus in parrot phylogenies. Although the taxonomic status of the genus *Aratinga* within *Arini* tribe was clarified in molecular studies, the relationships of the former *Aratinga* members still remain debatable and controversial. The most comprehensive and species-rich phylogenetic analysis of the *Arinae* subfamily, which has been performed so far, classifies the species *Aratinga* as sister to *Eupsittula* clade [4]. However, the node consisting of these two genera is supported only by a moderate Bayesian posterior probability. Other analyses showed that the species *Aratinga* are sister to the extinct Carolina parakeet [11], the species of *Eupsittula* [8] or macaws genera, such as *Ara*, *Cyanopsitta*, *Orthopsittaca* and *Primolius* [9, 12]. These discrepancies depend on the molecular markers used, among which the most popular are those derived from mitochondrial genomes. Thus, it is important to check whether mtDNA can reliably infer the species tree or phylogenies are affected by short length of sequences under study [11] and/or incompleteness of combined alignments [4].

Summarizing, the phylogenetic position of the present species *Aratinga* is still disputable and more molecular data are required to reconstruct their precise and well-resolved phylogeny. It was demonstrated that the complete mitochondrial genomes can provide useful information for the evolutionary studies of many taxa [13]. Hence, to obtain a better resolved phylogeny of *Arini* and solve the controversies about their relationships, we carried out comprehensive phylogenetic analyses based on the representative and complete mitochondrial genomes, applying various methodological approaches. We determined the usefulness and applicability of different phylogenetic methods and various molecular markers in the phylogeny of parrots at the tribe level. Even though various molecular markers (mostly *cox1*, *cytb* and *nd2*) have been used so far in inferring the phylogenetic relationships in parrots, including *Arini*, a variety of tree topologies have been produced [4, 8, 9, 11, 12]. The phylogenetic inconsistencies may result from various evolutionary rates of individual molecular markers and the quality of phylogenetic signal. Conserved markers can contain too low variation to provide a sufficient resolution of trees, whereas the phylogenetic signal in non-conserved sequences can be blurred because of multiple substitutions and homoplasy. The biased signal in individual mitochondrial markers was shown for metazoans [14], vertebrates [15–17], amniotes [18], insects [19], teleosts [20] as well as selected groups of amphibians [21, 22] and mammals [23–25]. Yet, such systematic studies were not undertaken for birds, although reconstruction of their phylogenetic relationships relied upon one or several, often arbitrarily selected, mitochondrial markers. This is why it is necessary to assess systematically the performance and usefulness of individual markers in phylogenetic studies of this group, and to verify if mtDNA phylogenies can credibly infer its species trees. To address these problems, we considered the case of the parrot tribe *Arini* and checked the consistency between the markers as well as how, on the strength of these markers, the phylogenies correspond with morphology.

## Methods

### Sequence data

The molecular markers under study were derived from ten complete mitochondrial genomes of the representative genera *Arini* (Table 1, see Additional file 1). We created two sets of taxa to ascertain also the influence of taxon sampling on the reconstructed phylogenetic relationships. The 7-taxa set included *Ara glaucogularis* and members of the former broadly defined genus *Aratinga*, namely *Aratinga solstitialis*, *Eupsittula pertinax*, *Phyrrhura rupicola*, *Psittacara mitratus* and *Thectocercus acuticaudatus*. The 11-taxa set contained in addition *Guaruba guarouba*, *Orthopsittaca manilata*, *Primolius couloni* and *Rhynchopsitta terrisi*. *Amazona barbadensis* from the closely related tribe *Androglossini* was used as an outgroup. Its inclusion allowed us to determine the branching order and the basal taxon within the ingroup *Arini*.

**Table 1:** Complete mitochondrial genomes of parrots analysed in the study

Species	Abbreviation	Accession	Length [bp]	Reference
*Amazona barbadensis*	Ab	JX524615	18,983	[84]
*Ara glaucogularis*	Ag	JQ782215	16,983	[85]
*Aratinga solstitialis*	As	JX441869	16,984	[62]
*Eupsittula pertinax*	Ep	HM640208	16,980	[50]
*Guaruba guarouba*	Gg	JQ782217	17,008	[86]
*Orthopsittaca manilata*	Om	KJ579139	16,985	[87]
*Primolius couloni*	Pc	KF836419	16,995	[88]
*Psittacara mitratus*	Pm	JX215256	16,984	[89]
*Pyrrhura rupicola*	Pr	KF751801	16,994	[90]
*Rhynchopsitta terrisi*	Rt	KF010318	17,027	[91]
*Thectocercus acuticaudatus*	Ta	JQ782214	16,998	[6]

**Fig. 1:**
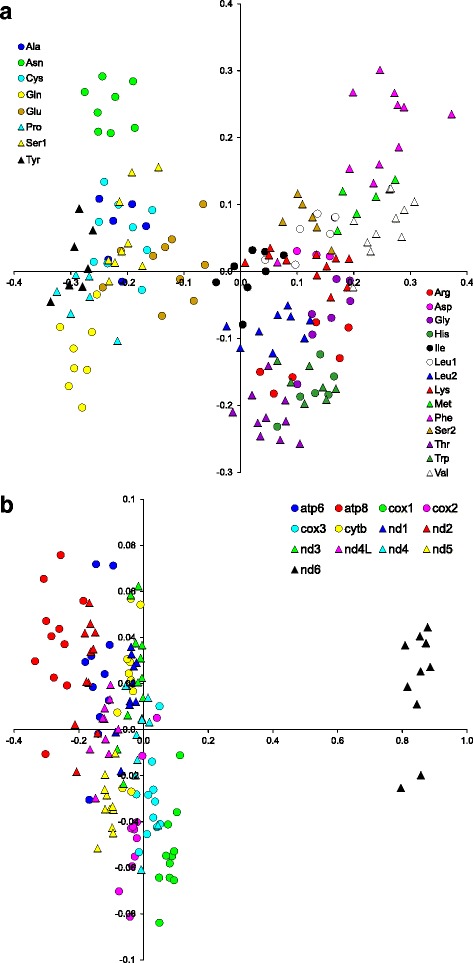
Correspondence analysis based on the nucleotide composition of tRNA (**a**) and protein-coding genes (**b**). The first (X axis) and the second (Y axis) dimensions explain 67.0% and 26.9% variance for **a** panel, and 97.1% and 1.9% variance for **b** panel, respectively

We analysed all mitochondrial markers, both separately and in concatenated sets: 13 protein-coding genes, 12S and 16S rRNA, 22 tRNA sequences and the control region (CR) (Additional file 2). Drawing on the correspondence analysis (CA) performed in Statistica [26], tRNA gene sequences were clustered into two sets according to their nucleotide composition, which was related with their location on heavy and light DNA strands (Fig. 1a). Finally, we investigated 18 single markers (or their groups in the case of tRNAs) as well as the concatenated set of all nucleotide markers (called ALL set) and the set of all markers, excluding the control region (called ALL-CR) – Additional file 2.

The homologous sequences were aligned in MAFFT v7.215 using slow and accurate algorithm L-INS-i with 1000 cycles of iterative refinement [27]. The alignments were then edited manually in JalView [28]. Sites suitable for phylogenetic studies from the alignments of rRNAs and CR were selected in GBlocks 0.91b [29]. To maximize the number of these sites, we applied less stringent criteria, i.e. smaller final blocks, gap positions within the final blocks and less strict flanking positions, as it is illustrated on the web site http://molevol.cmima.csic.es/castresana/Gblocks_server.html.

### Phylogenetic and statistical analyses

We applied eight phylogenetic approaches for nucleotide data sets: two Bayesian (BA) analyses in MrBayes [30] and PhyloBayes [31], two maximum likelihood (ML) methods in TreeFinder [32] and PAUP [33], as well as maximum parsimony (MP) and distance methods: neighbour joining (NJ), minimum evolution (ME) and weighted least squares (WLS), as implemented in PAUP [33].

The best fitting models were applied for particular alignments (Additional file 2). For each individual protein-coding gene, we used separate nucleotide substitution models for each codon position in MrBayes and TreeFinder. In the concatenated sets ALL and ALL-CR, all the first, second and third codon positions from 12 protein-coding genes (PCGs) were examined as three separate partitions. The three codon positions of *nd6* were considered as three additional separate partitions because the sequence of this gene showed a biased nucleotide composition in comparison to other PCGs (Fig. 1b), which was related to the location of these genes on the heavy and light DNA strands, respectively. The remaining five markers (two rRNAs, two tRNAs sets, CR) were also described by their own substitution models.

The best fitting nucleotide substitution models in PAUP analyses, were selected from 1624 models in jModelTest 2.1 [34], whereas in TreeFinder, the models were chosen according to Propose Model module in this program (Additional file 2). In MrBayes analyses, we applied mixed models rather than fixed ones to specify appropriate substitution models across the large parameter space [35], but the models describing heterogeneity rate across sites (invariant and discrete gamma) were adopted according to jModelTest (Additional file 2). In PhyloBayes, we applied CAT + GTR + Γ model for the individual PCGs, as well as ALL and ALL-CR data sets, whereas GTR + Γ model was adopted for the remaining nucleotide markers (Additional file 2). The CAT is an infinite mixture model accounting for site-specific amino-acid or nucleotide preferences and then appropriate for concatenated sequences with sites evolving in different substitution patterns [31]. When gamma-distributed rate variation across the sites was implemented, we approximated it by five discrete rate categories.

To reduce a potential compositional heterogeneity in sequences related with AT or GC bias, we recoded respective nucleotides for purines (R) and pyrimidines (Y) [36–38]. The RY-coding was applied for the ALL data set and the alignments were then analysed in MrBayes and TreeFinder, assuming the selection of both partitioned and not-partitioned sets. In MrBayes, we adopted the assumptions as described above, whereas in TreeFinder, we selected the two-state model for nucleotides (GTR2) with the best fitting assumptions for rate variation across sites, i.e. gamma and invariant models. We also performed analyses in PhyloBayes assuming CAT + Poisson + Γ and Poisson + Γ models. Moreover, we analysed the recoded alignments in PAUP using ML, WLS, ME, NJ and MP methods with Cavender’s and Felsenstein’s models [39] combined with gamma and invariant models (CF + Γ + I), because such variant appeared best fitting according to AIC (Akaike Information Criterion) [40], AICc (the corrected Akaike Information Criterion) [41] and HQC (Hannan–Quinn Information Criterion) [42] criteria for 7-taxa and 11-taxa sets.

In MrBayes analyses, two independent runs starting from random trees, each using four Markov chains, were applied. The trees were sampled every 100 generations for 10,000,000 generations. In the final analysis, we selected the last 22,360 to 78,090 trees, depending on the data set, that reached the stationary phase and convergence (i.e. when the standard deviation of split frequencies stabilized and was lower than the proposed threshold of 0.01). In PhyloBayes, the number of components, weights and profiles of the applied models were inferred from the data. Two independent Markov chains were run for 50,000 generations with one tree sampled for each generation. Depending on the data set, the last 5000 to 45,000 trees from each chain were collected to compute posterior consensus trees after reaching convergence, when the largest discrepancy observed across all bipartitions (maxdiff) was below the recommended threshold 0.1.

All possible tree topologies, corresponding to 945 unrooted and 10,395 rooted trees, were evaluated for the 7-taxa set in TreeFinder and the following PAUP approaches: maximum likelihood (ML), minimum evolution (ME), weighted least squares (WLS) and maximum parsimony (MP). For the 11-taxa set, we set the search depth at 2 in TreeFinder, whereas in PAUP, the final tree was searched from ten starting trees constructed by stepwise and random sequence addition, followed by the tree-bisection-reconnection (TBR) branch-swapping algorithm for ML, ME, WLS and MP methods. To assess the significance of the individual branches, non-parametric bootstrap analyses were performed on 1000 replicates in TreeFinder and PAUP. In the bootstrap analysis, we applied the branch-and-bound algorithm for the 7-taxa set and the TBR algorithm for the 11-taxa set in PAUP.

Topology tests were carried out in Consel [43] assuming 1000,000 replicates based on site-wise log-likelihoods calculated in TreeFinder under the best fitting substitution models for the ALL and ALL-CR data sets. We also compared competitive topologies in MrBayes using Bayes factor based on the stepping-stone method to estimate the marginal likelihood. In this approach, we assumed four independent runs, 50 steps of the sampling algorithm and 10,000,000 generations of the MCMC simulation. The distances between trees were calculated as symmetric difference of Robinson and Foulds in Treedist from Phylip package [44].

Codon-based tests of positive selection (Z-test and Fischer’s Exact test) as well as the average evolutionary divergence for the individual *Arini* markers were computed in MEGA package [45, 46]. The distance was based on Maximum Composite Likelihood method, and expressed by the number of nucleotide substitutions per site. Standard error estimate was calculated by a bootstrap procedure assuming 1000 replicates. Pearson correlation coefficients were determined in Statistica [26] between the mean tree distances for the individual markers and their lengths as well as the divergence. Additionally, we calculated Pearson coefficients correlating the number of clades with the assumed support thresholds and the two latter parameters.

## Results

### Evolutionary diversity of mitochondrial markers

**Fig. 2:**
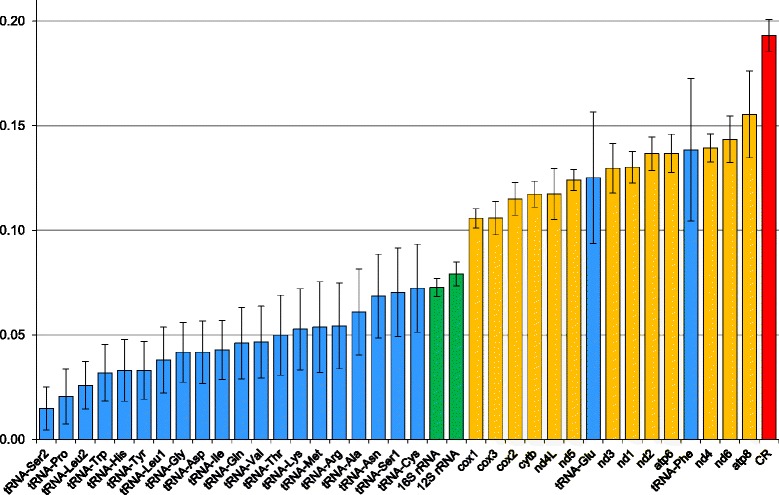
The average evolutionary divergence, i.e. the mean number of nucleotide substitutions per site, for individual sets of *Arini* markers. The whiskers indicate standard error calculated using the bootstrap approach

To compare evolutionary divergence among all mitochondrial markers of ten *Arini* representatives, we computed the mean number of nucleotide substitutions per site (Fig. 2). Generally, the majority of tRNA genes were characterized by a very low number of substitutions per site among the sequences under study (from 0.015 to 0.072). The most conserved were tRNA-Ser2 and tRNA-Pro. However, tRNA-Glu and tRNA-Phe, with the divergence of 0.13 and 0.14, clearly deviated from the other tRNAs. Two rRNA genes had the divergence of 0.073 and 0.079, close to the upper limit of the conserved tRNAs. Protein coding genes evolved with a rate generally greater than RNAs. Among PCGs, genes encoding three subunits of cytochrome c oxidase (*cox1*, *cox2*, *cox3*) showed the lowest divergence of about 0.11. On the other hand, the most divergent were genes coding for NADH dehydrogenase subunits (*nd4* and *nd6*) and ATP synthase F0 subunit 8 (*atp8*). These genes showed 0.14 and 0.16 substitutions per site, respectively. The control region (CR) was characterized by the largest divergence, i.e. 0.19.

### Comparison of phylogenies for individual mitochondrial markers

We obtained 144 phylogenetic trees for 18 markers using eight methodological approaches for each of the two data sets (Additional files 3 and 4). The trees with 7 taxa represented 72 different relationships and 39 topologies were inferred only once. Only two topologies were retrieved several times: one was obtained in five approaches for the control region as well as in one method for *atp6* and *nd2* (Additional file 3), the other was found by five approaches for *cox2* and two methods for *nd3*. For the 11-taxa set, 105 different topologies were produced and 81 of them were obtained only once (Additional file 4). In this case, only one topology dominated and was retrieved by seven approaches for the control region.

**Fig. 3:**
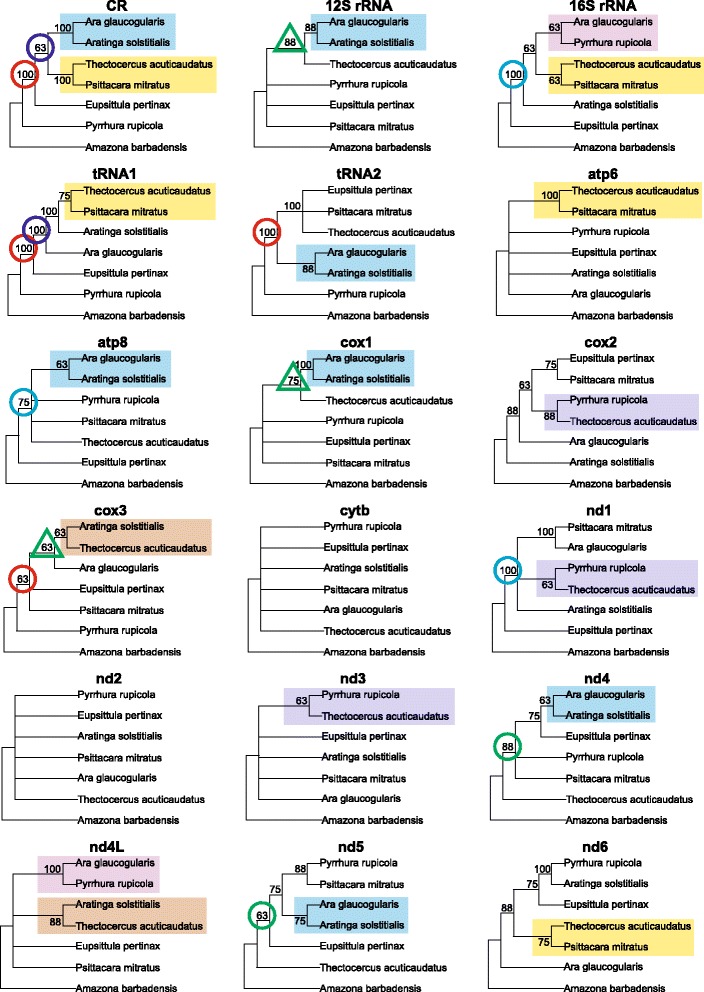
Consensus trees built employing eight methods for individual mitochondrial markers from 7 taxa parrots. Only clades supported in at least 50% of trees were shown. The same groupings of taxa are marked by the same colours or graphical symbols

**Fig. 4:**
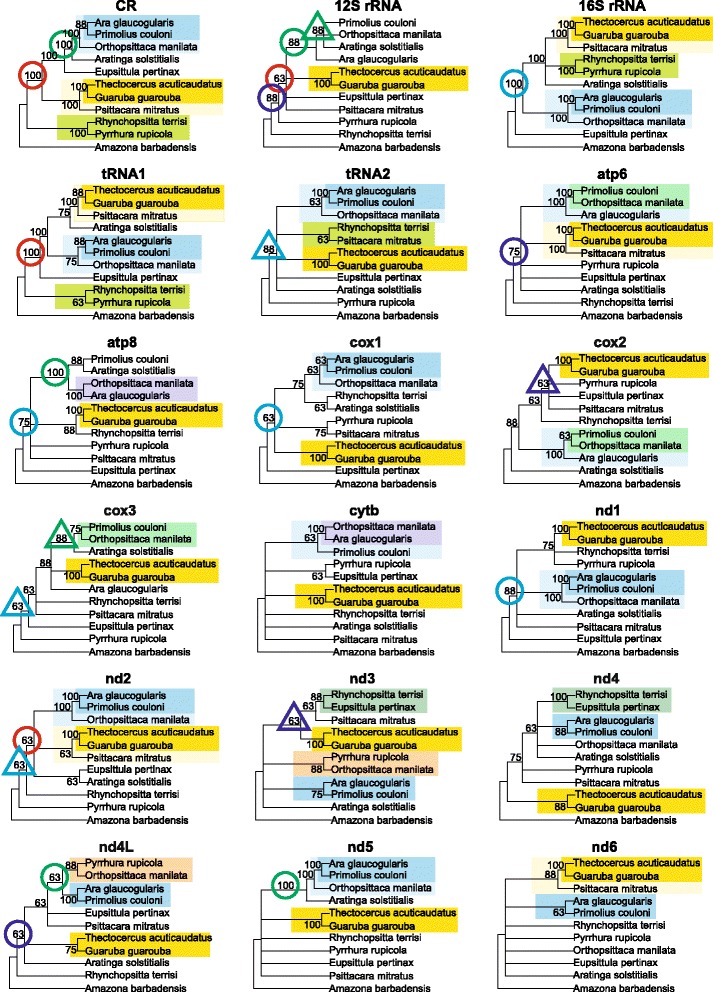
Consensus trees constructed using eight methods for individual mitochondrial markers from 11 taxa parrots. Only clades supported in at least 50% were shown. The same groupings of taxa are marked by the same colours or graphical symbols

To compile the results from various tree-building approaches for individual markers, we calculated for them the 50%-consensus from individual trees (Figs. 3 and 4). There was a rather weak consistency between the tree topologies inferred by various phylogenetic methods for the same molecular marker. For the 7-taxa set, the weakest agreement among these methods was for *cytb* and *nd2* genes, whose consensus trees were fully polytomous for *Arini* taxa (Fig. 3). In the case of *atp6* and *nd3*, only one clade was inferred by more than half of the methods. The fully resolved consensus trees were found for six markers. The number of clades that were predicted by all the methods was the highest for the CR and tRNA1 trees, which had three such clades out of four possible. In the case of the 11-taxa set, the greatest disagreement among the methods was for *nd6*, whose consensus tree had only three resolved clades out of possible eight (Fig. 4). The trees for *cytb* and *nd5* had half of such clades. Only two trees, for CR and tRNA1, had all the clades resolved. The trees based on CR and 16S rRNA had seven clades that were inferred by all the methods.

A lack of full concordance was also visible when we compared the consensus trees for individual markers (Figs. 3 and 4). All of the six fully resolved trees with 7 taxa were different (Fig. 3). It was possible to find competitive groupings that were obtained by all the eight methods, i.e. with 100% support, for different markers: for example, the clade *Aratinga solstitialis* + *Ara glaucogularis* occurred in the CR and *cox1* trees, and conversely, the clade clustering *Ara glaucogularis* with *Psittacara mitratus* was in *nd1* tree or with *Pyrrhura rupicola* in *nd4L* tree. The basal placement of *Pyrrhura rupicola* with other *Arini* was supported with 100% certainty in the CR and tRNAs trees, and likewise such basal location of *Eupsittula pertinax* was in the 16S rRNA and *nd1* trees. Nevertheless, there were some groupings that were present in several consensus trees for the individual markers (Fig. 3). The most frequently occurring clade grouped together *Aratinga solstitialis* and *Ara glaucogularis*. It was present in seven consensus trees for CR, 12S rRNA, tRNA2, *atp8*, *cox1*, *nd4* and *nd5* markers. The clade *Thectocercus acuticaudatus* + *Psittacara mitratus* was in the consensus trees for CR, 16S rRNA, tRNA1, *atp6* and *nd6*.

For the 11-taxa set, the clade *Thectocercus acuticaudatus* + *Guaruba guarouba* was present in all the 18 consensus trees, with 100%-support in 15 cases (Fig. 4). This clade, with the addition of *Psittacara mitratus*, occurred in six trees. The grouping of *Ara glaucogularis* + *Primolius couloni* was also frequently recovered in 12 consensus trees and was supported by all the methods in six trees. The clade containing these two taxa, as well as *Orthopsittaca manilata*, could be found in 11 consensus trees with the full support in six trees. However, deep bipartitions were much less reproducible and all the consensus trees with 11 taxa for the individual markers were unique (Fig. 4). Consequently, there were many mutually exclusive clades with the maximum support. For example, the clade including *Ara glaucogularis* + *Primolius couloni* was in the trees for 16S rRNA, tRNA1, *nd1*, *nd2*, *nd4L* and *nd5*, whereas *Ara glaucogularis* + *Orthopsittaca manilata* in trees for *atp8* and *cytb*, and *Orthopsittaca manilata* + *Primolius couloni* in *atp6* tree. The 100%-supported clade *Rhynchopsitta terrisi* + *Pyrrhura rupicola* occurred in CR and 16S rRNA trees and its alternative clade, *Rhynchopsitta terrisi* + *Eupsittula pertinax* in *nd4* tree. *Eupsittula pertinax* was at the base to other *Arini* taxa in the tree for 16S rRNA, whereas such position was occupied by *Rhynchopsitta terrisi* + *Pyrrhura rupicola* in CR and tRNA1 trees.

We also expressed the self-consistency of the applied phylogenetic approaches for individual markers by the mean of pairwise distances between the trees built by various phylogenetic methods. The distance was calculated as the symmetric difference of Robinson and Foulds (Table 2). The distance equal 0 indicates the same topology of compared individual trees, whereas the maximum possible value for the topology in question, with seven taxa, is eight and for eleven taxa is 16. For both the 7- and 11-taxa sets, the smallest distance was for the control region. Quite congruent topologies were also inferred for tRNA2 in 7-taxa sets and 16S rRNA in 11-taxa set. The most variable topologies were for *cytb* and *nd2* in 7-taxa set, and *nd6* in 11-taxa set.

**Table 2:** Mean distances calculated as the symmetric difference of Robinson and Foulds between trees constructed using different methods for particular markers

Marker	Data set	
	7 taxa	11 taxa
*12 s rRNA*	4.00	7.96
*16 s rRNA*	3.21	1.89
*atp6*	3.62	6.39
*atp8*	5.24	6.42
*cox1*	3.49	8.06
*cox2*	3.83	5.80
*cox3*	4.62	6.39
*CR*	1.07	0.50
*cytb*	5.86	8.25
*nd1*	2.96	6.07
*nd2*	5.76	6.57
*nd3*	5.29	8.32
*nd4*	3.79	6.47
*nd4L*	3.66	7.73
*nd5*	4.31	6.04
*nd6*	3.88	8.89
*tRNA1*	2.97	3.72
*tRNA2*	1.56	8.07

The minimum distance = 0 and maximum distance = 8 for 7-taxa set and 16 for 11-taxa set

One could assume that the consistency of tree topologies is related to the length and the evolutionary rate of molecular markers. However, the correlation between the mean tree distances for individual markers and their lengths was not statistically significant, although it was not unexpectedly negative, namely, the longer the sequence, the smaller the stochastic error: *r* = −0.270, *p* = 0.279 (for 7-taxa set) and *r* = −0.364, *p* = 0.137 (for 11-taxa set). Similarly, the correlation between the mean tree distances and the mean number of substitution per site for alignments of individual markers was low and not statistically significant: *r* = 0.165, *p* = 0.512 for 7-taxa set and *r* = −0.150, *p* = 0.553 for 11-taxa set.

To check if the different phylogenetic approaches can generate similar topologies, we calculated 50% consensus trees constructed by the individual methods for all markers. However, the consensus trees with 7 taxa were fully polytomous for almost every method used. Only the maximum parsimony method resolved two clades, but with the marginal support of ten markers out of 18. The consensus trees based on the 11-taxa set were also poorly resolved. Only three clades of closely related taxa were produced. *Thectocercus acuticaudatus* + *Guaruba guarouba* clade was recovered by all the methods with the support of 17 or 18 markers. The second most frequent clade of *Ara glaucogularis* + *Primolius couloni* was retrieved for 10 to 12 markers by all the methods, with the exception of maximum parsimony. The clade containing the above-mentioned two taxa and *Orthopsittaca manilata* did not appear in the consensus tree of minimum evolution and neighbour joining trees, and in others was supported by 10 to 13 markers. The other clades were not resolved. It seems to indicate that the phylogenetic signal in individual markers is extremely variable and that the impact of selecting a given tree-building method rather than another is negligible.

### Significance of phylogenies for individual mitochondrial markers

We also counted the number of clades in the trees for the individual markers, which were supported by posterior probability (PP) or bootstrap percentage (BP) for three levels of significance: PP > 0.70 or BP > 50%, PP > 0.95 or BP > 70% as well as PP > 0.99 or BP > 90%. Among all the 568 identified nodes for all the methods, all the markers and the 7-taxa set, there were 194 cases (34%) that satisfied the first level of significance. For the other two levels, the number of the supported nodes drastically decreased to 59 (10%) and 12 (2%), respectively. The inclusion of 11 taxa did not improve the support because for all 1152 possible nodes there were only 294 (26%), 128 (11%) and 69 (6%) cases for the three corresponding significance levels. The mean numbers of clades in trees based upon individual markers were presented in Table 3. For the topology in question with 7-taxa, there are four possible clades that can receive any support. The largest number of clades, i.e. 3.4 on average, with the first level of significance occurred for the trees based on the control region. The worst supported trees were reconstructed for *nd2* and *nd5* genes. More significantly supported clades (with PP > 0.95 or BP > 70%) occurred only for eight markers. The CR trees again had three such clades on average. However, highly supported clades with the third level of significance were found only in CR and tRNA2 trees. Similarly, the trees with 11 taxa based on only the control region contained a substantial number of significant nodes with PP > 0.70 or BP > 50%, i.e. 7.3 on average, out of eight possible nodes (Table 3). The worst markers for the first level of significance were *nd3*, *nd4L* and *nd6*. Nodes with PP > 0.95 or BP > 70% were absent from the trees based on *nd3*, *nd4L* and tRNA1. The highest level of significance was obtained usually by single nodes for the majority of markers, except for CR, whose trees had 3.6 such nodes on average.

**Table 3:** Mean number of clades with a given support, posterior probability (PP) or bootstrap percentage (BP) obtained by different methods for particular markers

Marker	7 taxa			11 taxa		
	PP > 0.70 or BP > 50	PP > 0.95 or BP > 70	PP > 0.99 or BP > 90	PP > 0.70 or BP > 50	PP > 0.95 or BP > 70	PP > 0.99 or BP > 90
*12 s rRNA*	1.88	0.00	0.00	3.38	1.38	1.00
*16 s rRNA*	0.88	0.38	0.00	4.25	1.25	1.00
*atp6*	0.63	0.00	0.00	2.50	1.25	0.38
*atp8*	1.13	0.00	0.00	2.88	1.13	0.13
*cox1*	1.25	0.34	0.00	2.50	1.13	1.00
*cox2*	1.25	0.00	0.00	1.75	0.75	0.38
*cox3*	1.25	0.00	0.00	2.13	1.13	0.63
*CR*	3.38	2.88	1.38	7.25	4.88	3.63
*cytb*	1.13	0.00	0.00	3.00	1.38	1.00
*nd1*	1.88	1.00	0.00	3.00	2.75	1.13
*nd2*	0.13	0.00	0.00	2.88	2.50	1.25
*nd3*	0.63	0.00	0.00	1.50	0.00	0.00
*nd4*	2.25	0.50	0.00	3.38	1.13	0.50
*nd4L*	1.13	0.50	0.00	1.00	0.00	0.00
*nd5*	0.25	0.00	0.00	4.25	2.38	1.25
*nd6*	0.63	0.00	0.00	1.63	1.00	0.88
*tRNA1*	2.63	1.13	0.00	2.75	0.00	0.00
*tRNA2*	2.00	0.63	0.13	3.63	1.38	0.38

The maximum number of possible clades that can receive any support is 4 for 7-taxa set and 8 for 11-taxa set

For the trees with 11 taxa, we found a significant correlation between the length of the markers and the number of the clades with the nodes supported by three levels of significance: PP > 0.70 or BP > 50% (*r* = 0.668, *p* = 0.002), PP > 0.95 or BP > 70% (*r* = 0.568, *p* = 0.014) and PP > 0.99 or BP > 90% (*r* = 0.610, *p* = 0.007). The correlation was also significant between the sequence divergence and the second (*r* = 0.510, *p* = 0.031) and the third level of significance (*r* = 0.481, *p* = 0.043). However, we did not observe such significant relationships for data with 7 taxa.

### Phylogenetic analyses based on concatenated sets of all markers in comparison with single markers

**Fig. 5:**
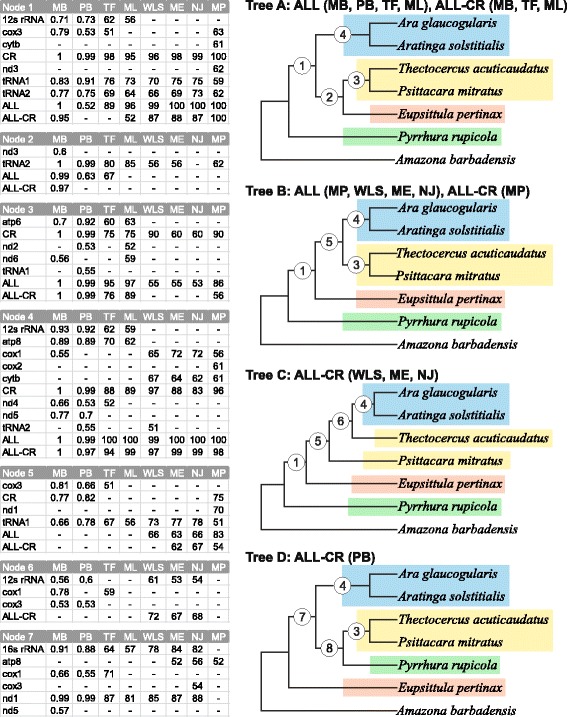
Tree topologies with 7 taxa (A, B, C and D) obtained from concatenated alignments including all markers (ALL) and without the control region (ALL-CR) for the following phylogenetic approaches: Bayesian in MrBayes (MB) and PhyloBayes (PB), maximum likelihood with partitioned data in TreeFinder (TF) and not-partitioned in PAUP (ML) as well as neighbour joining (NJ), minimum evolution (ME), weighted least squares (WLS) and maximum parsimony (MP) in PAUP. The abbreviations of the data sets and methods that produced the given topology are shown above the corresponding tree. Support values at individual nodes for various combinations of alignment sets and methods are presented in accompanying tables. The posterior probabilities ≤0.5 and bootstrap percentages ≤50 were indicated by a dash “-”. The node 8 received no support under these thresholds

Since the trees for individual markers were not fully resolved and showed contradictory relationships among the taxa under study, we carried out phylogenetic analyses using concatenated alignments of all markers to enhance the phylogenetic signal and reduce the stochastic error. The eight approaches produced only two tree topologies for 7 taxa, assigned as A and B in Fig. 5. The first one was favoured by two Bayesian methods in MrBayes and PhyloBayes as well as two maximum likelihood methods in TreeFinder and PAUP, whereas the second tree was produced by maximum parsimony and three distance methods, i.e. neighbour joining, minimum evolution and weighted least squares, in PAUP. In both trees, *Pyrrhura rupicola* had a basal position to the other *Arini* representatives, *Aratinga solstitialis* was grouped together with *Ara glaucogularis*, whereas *Psittacara mitratus* with *Thectocercus acuticaudatus*. The only difference between these topologies existed in the case of the placement of *Eupsittula pertinax*. In the topology A, it was sister to the clade of *Psittacara mitratus* + *Thectocercus acuticaudatus*, whereas in the topology B, it diverged before the split of *Aratinga solstitialis*, *Ara glaucogularis*, *Psittacara mitratus* and *Thectocercus acuticaudatus*.

**Fig. 6:**
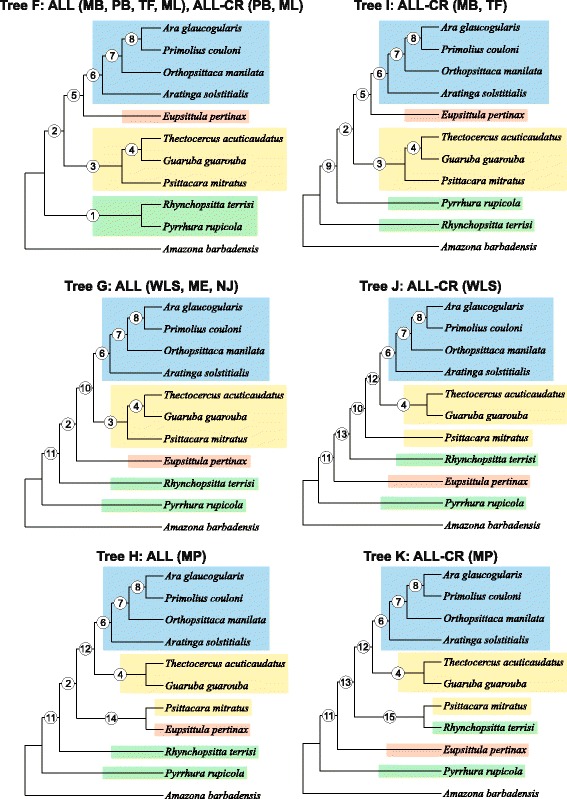
Tree topologies with 11 taxa (F, G, H, I, J and K) obtained from concatenated alignments including all markers (ALL) and without the control region (ALL-CR) for the following phylogenetic approaches: Bayesian in MrBayes (MB) and PhyloBayes (PB), maximum likelihood with partitioned data in TreeFinder (TF) and not-partitioned in PAUP (ML) as well as neighbour joining (NJ), minimum evolution (ME), weighted least squares (WLS) and maximum parsimony (MP) in PAUP. The abbreviations of the data sets and methods that produced the given topology are shown above the corresponding tree. Support values at individual nodes were presented in Fig. 7

**Fig. 7:**
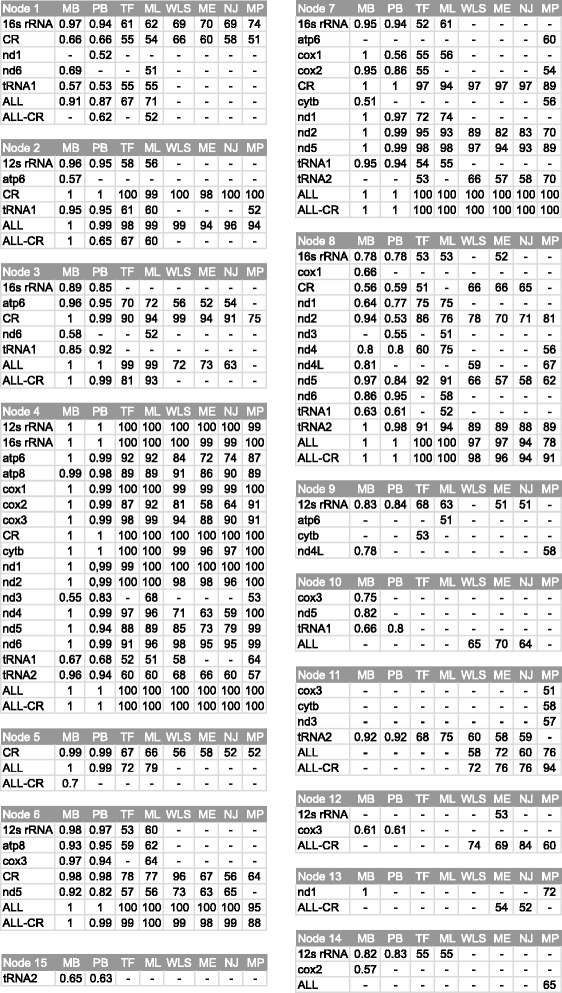
Support values at individual nodes for various combinations of alignment sets and methods for topologies with 11 taxa presented in Fig. 6. The posterior probabilities ≤0.5 and bootstrap percentages ≤50 were indicated by a dash “-”

For the concatenated alignment of all markers with 11 taxa, we arrived at three topologies called F, G and H (Figs. 6 and 7). Similarly to the 7-taxa set, topology F was obtained by Bayesian and maximum likelihood methods, whereas topology G by distance methods and topology H only by maximum parsimony. All the trees contained the clade including four genera: *Ara glaucogularis*, *Primolius couloni*, *Orthopsittaca manilata* and *Aratinga solstitialis* (APOA clade). The clade grouping together *Thectocercus acuticaudatus*, *Guaruba guarouba* and *Psittacara mitratus* (TGP clade) was present in F and G trees, but H tree exhibited only *Thectocercus acuticaudatus* + *Guaruba guarouba* grouping. All the trees differed in the location of *Eupsittula pertinax*. In tree F, it was grouped together with APOA clade, in tree G it was at the base to APOA + TGP clades and in tree H it clustered with *Psittacara mitratus*. Tree F showed also common origin of *Rhynchopsitta terrisi* and *Pyrrhura rupicola* lineages, which branched off successively at the base of other trees. Comparing trees from 7- and 11-taxa sets, it can be safely concluded that only tree B and G show corresponding topologies.

Having the topologies based on the most character-rich and presumably reliable set, it was interesting to check which individual markers and methods were capable of producing such topologies. Hence, we calculated the distances between these topologies and every tree constructed by the various methods for the individual markers. Unexpectedly, among all the 144 possible combinations of markers and methods, only nine trees with 7 taxa had the same topologies as the trees based on the concatenated set (Additional file 5). The trees built by three distance methods for the control region had the same topology as the tree A, whereas Bayesian, maximum likelihood and maximum parsimony topologies based on this marker were identical with the topology B. Interestingly, only MP tree for *atp6* showed the topology B. It is noteworthy that two tRNA sets for the many methods were capable of yielding trees quite similar, with the distance 2, to the topologies derived from all markers. The ML trees of *nd2* were also very close to the final topologies. Nonetheless, the most different topologies, with the distance 8, were very often generated because within the total of 18 markers 12 had at least two such trees. We did not notice any preference of methods in favour of such disparate topologies. Generally, the deviated trees were most often produced for *nd1*, *nd4L*, *nd3* and *cox2* sequences. Averaged for all the methods, their distance to the topologies based on the full-marker set was greater than 7.

The weak correspondence between the trees for individual markers and concatenated alignments appeared also for the trees with 11 taxa (Additional file 6). The trees exclusively based on the control region and produced by all the methods except for maximum parsimony were identical with tree F. Moreover, only tRNA1 tree inferred by weighted least squares method had the same topology as G tree. Quite small distances (2 and 4) to the F, G or H topologies were noticed in other tRNA1 trees and several trees for tRNA2, *nd2*, *nd5* and *atp6*. Still, the vast majority of the other trees based on individual markers were considerably different from those based on the concatenated alignment. Many topologies deviating from the expected topology were reconstructed by virtue of *atp8*, *cox1*, *cytb*, *nd3*, *nd4* and *nd4L* sequences. Such topologies were usually generated by distance methods.

### Phylogenetic analyses based on concatenated sets of markers without the control region

Since the control region alone was instrumental in recovering the tree topologies based on the concatenated sequences of all markers and in providing the best supported and consistent trees, we removed it from the concatenated alignment to find which phylogenetic signal lasted in the remaining markers. Surprisingly, we also obtained the same tree topologies as for the whole set. In the case of the 7-taxa set, the topology A was again supported by MrBayes and two maximum likelihood methods, whereas the topology B by maximum parsimony (Fig. 5). In addition, two other topologies appeared, assigned as C, supported by three distance methods, and D, produced by PhyloBayes. Both C and D indicated a close relationship between *Aratinga solstitialis* and *Ara glaucogularis*. The D topology contained the clade *Psittacara mitratus* + *Thectocercus acuticaudatus*, like the A and B topologies, but it was next joined to *Pyrrhura rupicola* placing *Eupsittula pertinax* basal with other *Arini*. The tree C maintained the early evolving *Pyrrhura rupicola* lineage, like A and B, but other taxa (*Eupsittula pertinax*, *Psittacara mitratus* and *Thectocercus acuticaudatus*) branched off successively and did not create clusters like in the other topologies.

Phylogenetic analyses of concatenated markers from 11 taxa, excluding the control region, produced four topologies (Fig. 6). One of them obtained by PhyloBayes and maximum likelihood method in PAUP was the same as the topology F for the full set of markers. The other topology named I, retrieved by MrBayes and TreeFinder, differed from F only in the separation of *Rhynchopsitta terrisi* and *Pyrrhura rupicola*. However, the other two topologies obtained by weighted least squares and maximum parsimony methods were markedly different. Not only did they separate *Psittacara mitratus* from *Thectocercus acuticaudatus* and *Guaruba guarouba,* but also placed *Eupsittula pertinax* between *Rhynchopsitta terrisi* and *Pyrrhura rupicola*. The trees for neighbour joining and minimum evolution methods were not fully resolved.

### Significance of phylogenies based on concatenated sets of markers

In Figs. 5, 6 and 7, we had additionally compiled support values for individual nodes of all ten topologies; these were based on concatenated sets of markers. Only posterior probabilities >0.5 and bootstrap percentages >50 were shown. The most firmly supported node 4, present in all four topologies with 7 taxa, joined *Aratinga solstitialis* and *Ara glaucogularis* (Fig. 5). This node corresponds with node 6 in topologies with 11 taxa (Figs. 6 and 7). These two nodes were supported with maximal or almost maximal posterior probabilities and bootstrap values for trees by all methods in both sets of the concatenated markers. Among individual markers, the control region supported this relationship quite significantly using a number of methods, too. Likewise, trees based on eight other markers from 7 taxa and four markers from 11 taxa corroborated this grouping for many phylogenetic approaches. The relationships within the clade converging to the node 6 in topologies with 11 taxa were very well resolved (Figs. 6 and 7). The same relationships were recovered in all topologies with maximal and very large support values not only in the trees based on concatenated sets but also on several individual markers. Interestingly, node 8 was only weakly supported by trees based on the control region in contrast to other markers, e.g. tRNA2.

The viability of node 3 (*Psittacara mitratus* + *Thectocercus acuticaudatus*) occurring in topologies A, B and D with 7-taxa (Fig. 5) and the corresponding node 3 in topologies F, G and I with 11-taxa (Figs. 6 and 7) was confirmed by all methods for the ALL set; only Bayesian and maximum likelihood methods produced very significant support. Again, trees for all the methods using the CR alignment contained this bipartition with moderate-to-high support. Curiously, three other markers including these nodes were the same in two sets with different number of taxa. In trees with 11 taxa, the clade supported by the node 3 included also *Guaruba guarouba*, which was clustered with *Thectocercus acuticaudatus* (Figs. 6 and 7). This grouping showed the largest support across almost all individual markers.

The node 1 separating *Pyrrhura rupicola* and other *Arini* was also present in three topologies with 7 taxa: A, B and C (Fig. 5). It was very well supported by all methods (except PhyloBayes tree) for the ALL set. All methods for the control region and two tRNA alignments also favoured this clade, the former very significantly and the latter moderately. This node corresponds to node 2 in topologies F, G, H and I with 11 taxa, which also enjoyed a very high support from the set based on all markers and the control region (Figs. 6 and 7). In topology F, *Pyrrhura rupicola* was grouped together with *Rhynchopsitta terrisi*, which was moderately and weakly supported by Bayesian and maximum likelihood methods for the ALL set. Such a cluster appeared in trees based on five individual markers, too. Five other topologies with 11 taxa had these two species separated. In topologies G, H, J and K, *Pyrrhura rupicola* lineage diverged earlier than *Rhynchopsitta terrisi* and in topology I the reverse was true. Such arrangements, though, claimed weaker support than the clade grouping these species.

The position of *Eupsittula pertinax* was the least stable. In topology A with 7 taxa, it grouped together with *Psittacara mitratus* + *Thectocercus acuticaudatus* in node 2, which was supported by posterior probability from 0.97 to 1 in the MrBayes trees for the ALL and ALL-CR sets as well as the MrBayes and PhyloBayes trees for tRNA2 and some other methods (Fig. 5). However, such placement of *Eupsittula pertinax* was not accomplished in any trees with 11 taxa. In trees F and I based on Bayesian and maximum likelihood methods, this species was most significantly clustered with the clade of *Ara*-*Primolius*-*Orthopsittaca*-*Aratinga*: node 5 in Figs. 6 and 7. This node had very high posterior probabilities and moderate-to-weak bootstrap values in trees based on the control region and ALL set. In tree B and C (node 5), *Eupsittula pertinax* was sister to *Aratinga solstitialis*, *Ara glaucogularis*, *Psittacara mitratus* and *Thectocercus acuticaudatus*, making the last four taxa monophyletic. Such position was supported by, for instance, maximum parsimony and distance methods for the concatenated sets and all trees based on tRNA1. Their monophyly was also present in topologies G and J but was poorly supported. Other nodes were very weakly supported or did not gain significant posterior probability or bootstrap percentages and can be disregarded.

### Topological test of *Arini* phylogenies

**Fig. 8:**
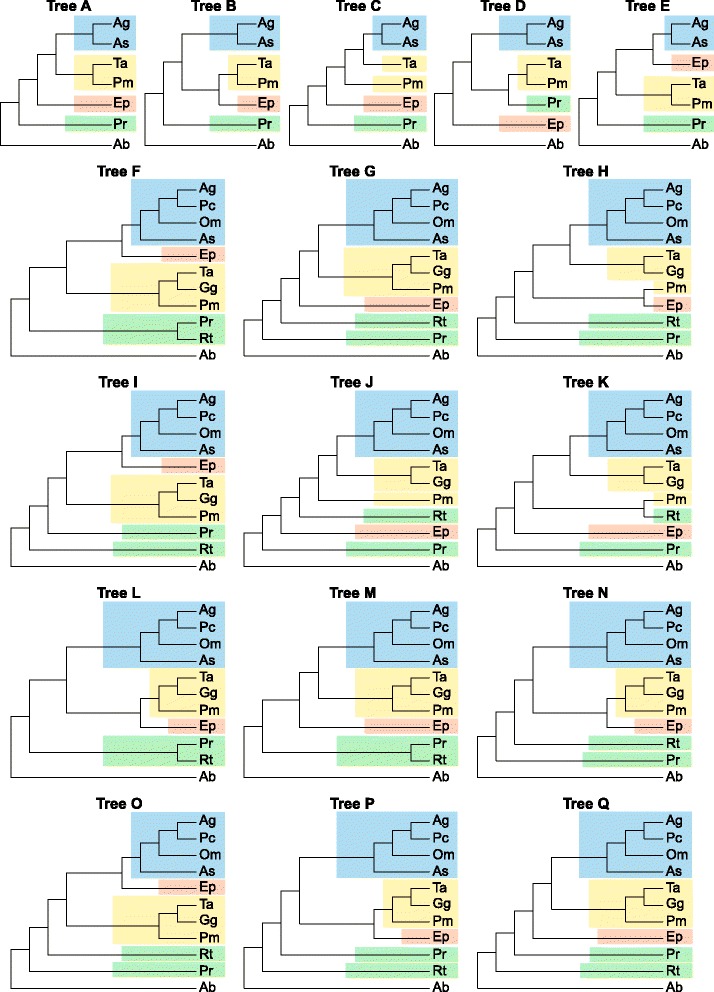
Tree topologies obtained for concatenated sets of markers and their alternatives compared in topological tests. Abbreviations of taxa names are explained in Table 1

We carried out six topological tests for the ALL and ALL-CR data sets to assess the statistical significance of the differences between the best found tree and competitive topologies with 7 and 11 taxa (Fig. 8, Table 4). The topology A was the best for the ALL and ALL-CR sets and was significantly better (with *p*-value <0.05) in four cases than topologies B, D and E. Topology C fared poorly, compared with A in ten cases. Similarly, Bayes factor test also demonstrated substantial differences between the topology A and other topologies (Table 5). A log difference in the range of 3–5 is typically considered to be a strong evidence in favour of the better hypothesis and a log difference larger than 5 is regarded as a very strong significance [47]. For the ALL set, the difference exceeded 5 when comparing tree A with others, whereas for the ALL-CR set, the difference turned out to be above 3 when comparing A with B and C.

**Table 4:** Results of tests (*p*-values) comparing the best tree topology with alternatives for data sets with 7 and 11 taxa including all mitochondrial markers (ALL) and excluding the control region (ALL-CR)

Data set	Tree	ALL						ALL-CR					
		AU	NP	BP	PP	SH	wSH	AU	NP	BP	PP	SH	wSH
7 taxa	A	0.892	0.756	0.756	0.995	0.942	0.965	0.841	0.680	0.680	0.986	0.930	0.917
	B	0.257	0.105	0.105	**0.003**	0.525	0.486	0.183	**0.037**	**0.037**	**0.003**	0.434	0.414
	C	**0.008**	**0.004**	**0.004**	**6.0E-12**	**0.025**	**0.029**	**0.020**	**0.009**	**0.009**	**1.0E-08**	0.050	0.059
	D	0.075	**0.039**	**0.039**	**2.0E-06**	0.184	0.161	0.303	0.229	0.229	**0.006**	0.450	0.457
	E	0.225	0.096	0.096	**2.0E-03**	0.501	0.447	0.171	**0.045**	**0.045**	**0.004**	0.449	0.421
11 taxa	F	0.833	0.500	0.495	0.900	0.976	0.989	0.552	0.160	0.158	0.194	0.926	0.921
	G	0.133	**0.014**	**0.014**	**5.0E-05**	0.525	0.501	0.386	0.056	0.055	**0.044**	0.883	0.882
	H	**0.004**	**0.001**	**0.001**	**7.0E-15**	**0.031**	**0.022**	0.108	0.056	0.055	**6.0E-06**	0.310	0.303
	I	0.428	0.150	0.150	0.062	0.820	0.725	0.603	0.188	0.185	0.231	0.942	0.946
	J	**1.0E-07**	**4.0E-08**	**0.000**	**8.0E-38**	**2.0E-05**	**3.0E-05**	**0.001**	**1.0E-04**	**1.0E-04**	**1.0E-15**	**0.008**	**0.009**
	K	**5.0E-07**	**5.0E-08**	**3.0E-07**	**9.0E-42**	**6.0E-06**	**3.0E-05**	**0.001**	**1.0E-04**	**1.0E-04**	**4.0E-16**	**0.007**	**0.016**
	L	0.323	0.101	0.101	**0.002**	0.671	0.616	0.498	0.113	0.110	0.095	0.894	0.900
	M	0.326	0.095	0.094	**0.002**	0.702	0.643	0.436	0.080	0.078	0.066	0.889	0.885
	N	0.113	**0.009**	**0.009**	**4.0E-05**	0.497	0.474	0.398	0.054	0.053	0.058	0.886	0.896
	O	0.319	0.085	0.084	**0.034**	0.809	0.660	0.536	0.132	0.129	0.163	0.935	0.924
	P	0.172	**0.025**	**0.024**	**9.0E-05**	0.530	0.516	0.479	0.104	0.102	0.089	0.888	0.882
	Q	0.180	**0.027**	**0.026**	**1.0E-04**	0.553	0.536	0.411	0.076	0.075	0.061	0.880	0.859

The topologies were presented in Fig. 8. Underlined letters and numbers refer to the best topology for the given data set. The following tests were used: approximately unbiased (AU), bootstrap probability calculated from all sets of the scaled replicates (NP), bootstrap probability calculated from one set of replicates (BP), Bayesian posterior probability calculated by the BIC approximation (PP), Shimodaira-Hasegawa (SH) and weighted Shimodaira-Hasegawa (wSH). Values smaller than 0.05 are shown in **bold**

**Table 5:** Results of Bayes factor test (expressed as the difference in log likelihood units) comparing the best tree topology with alternatives for data sets with 7 and 11 taxa including all mitochondrial markers (ALL) and excluding the control region (ALL-CR)

Data set	Tree	ALL	ALL-CR
7 taxa	A	0.0	0.0
	B	**5.6**	**3.1**
	C	**24.9**	**15.8**
	D	**13.1**	2.5
	E	**5.8**	2.9
11 taxa	F	0.0	0.2
	G	**9.4**	2.2
	H	**33.6**	**12.3**
	I	1.1	0.0
	J	**86.0**	**35.8**
	K	**94.6**	**35.6**
	L	**5.8**	**3.04**
	M	**5.7**	2.7
	N	**10.7**	2.2
	O	**3.3**	1.7
	P	**9.9**	2.3
	Q	**9.0**	2.8

The topologies were presented in Fig. 8. Zero refers to the best topology. Values larger than 3 are shown in **bold**

The best topology with 11 taxa for the concatenated alignments of all markers was F. It appeared significantly better than topologies L, M and O in one test, G, N, P and Q in three tests, and H, J, K in all six tests (Table 4). No significant difference was found only between topology F and I, which differed in position of *Pyrrhura rupicola* and *Rhynchopsitta terrisi* (Fig. 8). The results are compatible with Bayes factor test, which also showed a significant difference between topology F and other topologies, with the exception of topology I (Table 5). This topology was in turn the best one for the ALL-CR set and revealed a significant difference from topologies G and H in one test as well as J and K in all six tests. The common feature of topologies H, J and K was the disruption of monophyly of *Psittacara-Guaruba-Thectocercus* (Fig. 8). These topologies were also substantially worse than topology I by virtue Bayes factor test (Table 5). Topology L was marginally worse than I with Bayes Factor 3.04.

The best topologies F and I for the 11-taxa set corresponded roughly to the alternative topology E for the 7-taxa set, whereas the best topology A with 7 taxa was represented by the alternative topologies G, M or Q in the 11-taxa set. The differences between these best topologies and the alternatives representing the best tree from the data set with different number of taxa were statistically significant at least for the concatenated alignment of all markers.

### Phylogenies based on recoded nucleotides sequences

**Fig. 9:**
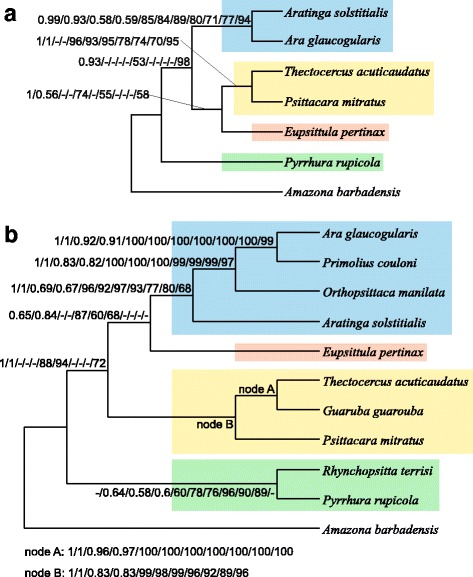
Tree topologies obtained for the ALL data set subjected to RY-coding for 7- (**a**) and 11-taxa set (**b**). Numbers at nodes correspond to support values calculated using the following methods: MrBayes under partitioned and not-partitioned model, PhyloBayes assuming CAT and Poisson model, TreeFinder under partitioned and not-partitioned model, maximum likelihood, weighted least squares, minimum evolution, neighbour joining and maximum parsimony in PAUP. The posterior probabilities ≤0.5 and bootstrap percentages ≤50 were indicated by a dash “-”

Since a compositional bias related with A + T content may contribute to artificial relationships [36–38], we recoded respective nucleotides for purines and pyrimidines in the alignment, including all mitochondrial markers to eliminate the potential compositional heterogeneity. The applied eleven approaches produced three trees with 7 taxa and five trees with 11 taxa. However, only one tree for each of these data sets received significant support (Fig. 9). The tree with 7 taxa (Fig. 9a) was produced by four approaches (partitioned and non-partitioned in MrBayes, non-partitioned in TreeFinder and maximum parsimony) and is identical with the topology A, which was found for the not-recoded sequences. The tree with 11 taxa (Fig. 9b) was reconstructed by five approaches (non-partitioned in MrBayes and TreeFinder, maximum likelihood, weighted least squares and minimum evolution in PAUP) and is identical with the topology F, which was also found by the largest number of methods for not-recoded sequences. Other trees based on the recoded data differed in the location of *Eupsittula pertinax* and *Pyrrhura rupicola* and/or broke the monophyly of *Pyrrhura rupicola* and *Rhynchopsitta terrisi*. However, the conflicting clades did not get posterior probabilities >0.5 or bootstrap percentages >50 and any supported clades in these trees were also present in the highly supported trees. Only a moderately supported bipartition with 67% bootstrap value was obtained for the 11-taxa set by maximum parsimony and separated *Amazona barbadensis* and *Rhynchopsitta terrisi* from other studied taxa.

## Discussion

### Sequence variation of mitochondrial markers

Our study showed that tRNAs are on average the most slowly evolving genes, which may be attributed to the strong constraints on their structure and function. Sequences of rRNA genes changed on average 1.7 times faster, protein-coding genes 3.1 times and the control region showed the greatest rate, which was 4.4 times higher than tRNAs. Yet, there were two outstanding genes, tRNA-Glu and tRNA-Phe, that showed divergence 3.1 times greater than other tRNAs in *Arini* mitogenomes. Two variable tRNA genes, albeit for other amino acids (Val and Ser), were found in the genomes from Epinephelidae fishes [48]. The order of the genes according to their evolutionary divergence is similar to the results calculated for mitochondrial genes in mammals [49] and birds, including parrots [50]. However, in the last group, there are some genes (*cox1* and *cox3*) that evolve more slowly than rRNAs. In our analyses of *Arini* members, all PCGs showed greater substitution rates than rRNAs. The studies of avian mitogenomes [50] seem to be in agreement with our findings: the most conserved PCGs are the two genes for cytochrome oxidase subunits I and III. The next conserved protein gene in *Arini* mitochondrial genomes was *cox2*, which, however, demonstrated much greater substitution rate in the global studies of bird and parrot mitogenomes [50]. The most diverged gene in *Arini* mitochondrial genomes was *atp8* and *nd6* was next line. In the global analyses of Aves and Psittaciformes [50], the most rapidly evolving gene was *nd2* and next *atp6*, which in *Arini* did not show the widest variation. The discrepancy is attributable to heterogeneous evolutionary rate of genes in time and various lineages, which should be included in global phylogenetic studies using members of different groups. Our study took into account short evolutionary distances (about 14 million years), whereas Pacheco et al. [50] studied a much longer period: 85 million years. The substitution rate of some genes may slow down or accelerate in the tribe *Arini*, but on a global scale we can observe an opposite effect. In agreement with that, *atp8* showed the least clock-like behaviour and was not the fastest evolving gene in the avian study [50] but it demonstrated the greatest variation in the *Arini* mitogenomes.

### Performance of individual mitochondrial markers in inferring phylogenies

Using data for the complete mitochondrial genomes in the representatives of the main evolutionary lineages of *Arini*, we tried to infer their phylogenetic relationships using individual mitochondrial molecular markers as well as concatenated sets. The results for some markers were, however, not satisfactory. The trees based on the same marker and obtained by various methods most often showed disparate evolutionary histories. Similarly, the same method applied to the various markers also produced different phylogenetic relationships. It should be emphasized that it was possible to find conflicting groupings of the same taxa that were supported by all methods for various markers. It implies that inferring phylogenetic relationships based on individual mitochondrial markers can lead to wrong and contradictory conclusions. The individual trees were usually characterized by poor resolution. Generally, only one tenth of all identified nodes for all the methods and all the markers had posterior probability >0.95 or bootstrap percentage > 70%, both for 7- and 11-taxa sets.

The sharpest disagreement of methods was registered for *cytb*, *nd2* and *nd6* genes. The trees based on *nd2*, *nd3*, *nd4L*, *nd5* and *nd6* belonged also to the worst resolved. It is remarkable that *cytb* and *nd2* markers are very often used in phylogenetic analyses, including parrots [4, 9, 11, 12]. Therefore, their results should be treated with caution. The most similar trees produced by different methods were for CR and next for tRNA and 16S rRNA genes. The trees constructed on the control region contained the largest number of well resolved nodes, too. The bias of individual markers in inferring phylogenetic relationships may be the effect of systematic and stochastic errors, too poor phylogenetic signal, unequal and differential substitution rate as well as the convergent changes in unrelated lineages resulting from similar mutational or selection pressures [21, 51–58].

The variable phylogenetic signal in various mitochondrial markers demonstrated in our study explains the inconsistent or unresolved phylogenies of *Arini* members obtained previously for various markers. For example, the tree based on *cox1* and *nd2* [11] and enhanced by two nuclear introns (*trop* and *tgfb2*) and coded gaps [12] grouped *Aratinga solstitialis* and *Ara glaucogularis* together in one clade, whereas in the tree based on *cox1*, *cytb*, *nd2* and *rag-1*, *Aratinga solstitialis* was clustered with *Eupsittula pertinax* [4]. The weak or conflicting phylogenetic signal in the used markers produced poorly or not significantly resolved relationships between other *Arini* members in the trees [4, 6, 9, 11, 12].

Contradictory tree topologies obtained from various mitochondrial markers were reported also for various groups of animals [14–25]. It could be hypothesised that the consistency of tree topologies is related to the length of molecular markers. Longer sequences with stronger phylogenetic signal could cause smaller stochastic error and trees based on them can show similar topologies regardless of the method used. Similarly, the level of agreement among tree topologies for individual markers could also depend on their evolutionary divergence. One could expect that the trees based on more conserved sequences are more stable and concordant, whereas highly variable sequences may lead to phylogenetic artefacts, which may be emphasized by some methods (e.g. long-branch attraction by maximum parsimony). On the other hand, less variable sequences may be affected by a poor phylogenetic signal and generate stochastic error. Nevertheless, our analyses demonstrated that the inconsistency and efficiency in recovering the correct nodes did not depend on the length and evolutionary rate of the markers [17, 18, 22]. Our results also revealed that the consistency of the trees computed using various methods did not significantly correlate with the length of the individual markers; on the other hand, we found a significant positive correlation between the resolution of trees, measured by number of supported nodes, and the divergence of markers measured by the mean number of substitution per site. This implies that the resolution of trees at the parrot tribe level may be improved by using fast evolving markers with sufficient number of variable and informative sites such as the control region.

The usage of single markers was also criticized because their phylogenies did not match those based on many markers or entire mitochondrial genomes [24, 59]. For example, the commonly used control region did not allow well resolved trees and failed to identify unique mitochondrial lineages in contrast to complete mitogenomes [59]. The marker selection and their number have also an influence on the molecular time estimation as it was proved for birds [60]. Generally, *cox1*, *nd1*, *nd2* and the concatenated RNAs can provide molecular time estimates most similar to those based on the complete mitogenomes. The most informative markers or group of markers needed to reconstruct reliable phylogenies vary with groups and should be selected individually not only for big lineages [14] but even for a given family or genus [23].

On the other hand, Seixas et al. [61] showed that full mitogenomes cannot provide better resolved phylogenies than single genes, which contrasts with other studies [24, 59]. The usefulness of single or numerous concatenated markers depends probably on the group under study and its level of evolutionary divergence as well as on the differences in the evolutionary rates of individual markers. For larger evolutionary distances, analysed by e.g. Seixas et al. [61], there is a larger probability that individual genes will display differentiated evolutionary patterns. Therefore, the combined contradictory signals can produce a noise rather than enhance the phylogenetic signal. However, for closely related taxa, such as those dealt with in this study, the phylogenetic signal is not so variable and the inclusion of many markers decreases the stochastic error, yielding more phylogenetic information. To alleviate the effect of the differentiated evolutionary rate of concatenated markers, it is important to apply partitioned models assuming separate substitution patterns and rates for individual markers and codon positions in protein-coding genes. In agreement with that, we found that more sophisticated methods, i.e. Bayesian and maximum likelihood assuming partitioned models produced trees with large support values for the concatenated alignments more often than simpler methods.

Nevertheless, it is difficult to indicate an individual marker that could reliably recover the phylogenetic relationship within *Arini* although the strongest phylogenetic signal was included in the control region and tRNA genes. The trees based on these markers were identical or very similar to the final topologies inferred from all markers for some methods. Interestingly, these two types of markers are characterized by opposite evolutionary rates in mitochondrial genomes. The former is rapidly evolving, whereas the latter is conserved on average. The usefulness of tRNA genes was usually underestimated. They were not commonly used in the reconstruction of phylogenetic relationships although their usefulness was also proved for bony fishes [20] and amphibians [22]. Nevertheless, neither CR nor tRNAs may be used alone because not all the nodes based on these markers were significantly supported and the topologies depended on the method used.

Our results indicate that the most reliable phylogenetic relationship within *Arini* may be obtained on the data sets consisting of concatenated alignments of at best all mitochondrial markers. The exclusion of the control region, which provided alone the strongest phylogenetic signal, did not remove vital information about the phylogenetic relationships. The most robust methods, Bayesian and maximum likelihood, were able to recover the same tree topology as for the complete data set. It is suggestive of sufficient information also present in other mitochondrial markers.

### Which phylogenetic relationships within *Arini* are reliable?

To infer phylogenetic relationships within *Arini* we applied eight methods on two data sets with 7 and 11 taxa to verify also the influence of the taxa number. The resulting topologies showed consistently the presence of two highly supported clades: (1) *Aratinga solstitialis* + *Ara glaucogularis* with the addition of *Primolius couloni* and *Orthopsittaca manilata* in the case of 11-taxa set, and (2) *Psittacara mitratus* + *Thectocercus acuticaudatus* with the addition of *Guaruba guarouba* in the 11-taxa set. The clades and the relationships within them were strongly supported and occurred also in many consensus topologies derived from individual trees constructed utilising various methods for individual markers.

Such groupings are supported by some common features present in mitochondrial genomes of the *Arini* [62] (see Additional file 1). Three species in the first clade share the same stop codons in *nd5* gene, whereas all members of the second clade have the same starts and stops in all genes. Two members of the first clade have one-nucleotide spacer between tRNA-Val and 16S rRNA genes, whereas two members of the second clade share exclusively a two-nucleotide spacer between tRNA-Ala and tRNA-Asn genes. All species in the second clade have the longest 12S rRNA gene (more than 975 bp) of all studied *Arini*.

The trees based on all markers also indicated an early divergence of *Pyrrhura rupicola*. In the case of 11-taxa set, this species clustered with *Rhynchopsitta terrisi* in all Bayesian and maximum likelihood trees based on all markers and almost all trees based on RY-recoded alignment. This grouping obtained higher statistical support than the separation of these species. Therefore, the monophyly of these genera seems to be the most probable. They also share the longer C-box (a conserved motif in the control region), whereas other *Arini* have a deletion (see Additional file 1). In support of the uniqueness of this lineage and the close relationship of other *Arini* members, *Pyrrhura* and *Rhynchopsitta* genomes are characterized by the lowest and highest A + T content, as well as the most and the least biased DNA symmetry (skew) among the *Arini* under study (see Additional file 1).

Among the inferred relationships, the position of *Eupsittula pertinax* is controversial. In the most probable tree for 7-taxa set (topology A), this taxon grouped together with the clade *Psittacara mitratus* + *Thectocercus acuticaudatus*. This tree was inferred on the set of all markers by powerful methods such as Bayesian and maximum likelihood, in contrast to maximum parsimony and distance methods, which favoured the alternative topology B. The topology A, which showed also greater support values for the conflicting clade, was significantly favoured by topology tests and was recovered on the alignment that was subjected to RY-recoding to reduce compositional bias. On the other hand, in the tree F, based on 11-taxa set and also supported by the powerful methods on the set of all markers and the RY-recoding alignment, *Eupsittula* was sister to the clade including *Ara glaucogularis*, *Primolius couloni*, *Orthopsittaca manilata* and *Aratinga solstitialis*. This clustering was also statistically more significant in support values and topological tests than alternative placements of *Eupsittula*. Tree A and F, including these two groupings, were much more probable than other competing topologies. These results illustrate that taxon sampling may influence tree topology. It is difficult to point to the most probable relationship on the basis of these data because support values for them are comparable. The topology F seems to be more probable because it is based on the set containing a larger number of taxa. The difficulties in determining phylogenetic relationships within *Arini* may result from a rapid divergence and differentiation of its members, as evidenced by short internal branches in phylogenetic trees.

As far as mitochondrial phylogenies are considered, we should be aware that mtDNA might not reflect the true species trees. The disagreement between the mtDNA and species tree may stem from introgression and incomplete lineage sorting following recent speciation [63]. However, these processes do not have to occur in birds. Following Haldane’s rule [64], the introgression of maternally inherited mtDNA is restricted between heterogametic avian species because female hybrids are characterized by a reduced viability [65–70]. Nevertheless, according to Rheindt and Edwards [71], it is true only for loci that are not subjected to natural selection. If the premise of neutrality is violated, the positive selection can lead to mtDNA introgression. Therefore, we tested the hypothesis about the positive selection for each of 13 protein-coding genes from the 11-taxa set, using Z-test and Fischer’s Exact test with all available models in the MEGA package [46]. In each case, we found that the null hypothesis of neutrality cannot be rejected and the alternative hypothesis of the positive selection should not be accepted with *p*-value = 1. It suggests that a sufficient number of non-synonymous substitutions did not accumulate during the evolution of the tribe *Arini* to cause the selection-dependent introgression.

Since mitochondrial markers are single-copy genes, they can be less susceptible to the incomplete lineage sorting than the nuclear ones. The nuclear genes more often duplicate and their different copies can disappear in various lineages, which can lead to hidden paralogy and disagreement between gene and species trees [54, 72–79]. However, the incomplete sorting may influence mtDNA if multiple population divergences or speciation events were closely spaced in time [80], which cannot be definitely excluded for the *Arini* taxa studied here.

The application of nuclear markers could provide additional information about phylogenetic relationships among parrots. However, the resolution of phylogenetic relationships based on available nuclear markers is much worse than mitochondrial ones [9, 81, 82]. For example, an alignment of nuclear RAG1 gene sequences from 36 *Arini* representatives has only 3.6% of variable sites (98/2544 bp), whereas an alignment based on mitochondrial *nd2* sequences from the same taxa has 49.9% (446/894 bp) of variable sites.

### Comparison of phylogenetic relationships with morphological data

Even a well-resolved phylogenetic tree does not have to reflect true relationships between the taxa under study, i.e. the species tree. To check to what extent the mtDNA phylogenies may correspond to the real *Arini* relationships, we confronted the phylogenetic results with the morphological data. Curiously, drawing on the molecular markers the proposed most probable relationships do not contradict the classification based on the colouration of the wings and tail, which were believed by Remsen et al. [5] to discriminate the new genera of this tribe.

Greater primary coverts of *Aratinga* are violet blue and its tail is olive-green tipped violet [5]. The sister taxon to *Aratinga* is *Ara*, which shows greater differentiation. Apart from four species that are predominantly green, two others are mostly blue and yellow [2]. Two additional species are characterized by a domination of red colouring [2]. However, greater primary coverts of all the eight *Ara* species are blue and the tail is also blue-tipped, regardless of interspecies differences in rectrices colouration [2]. *Primolius* have also blue colouration of greater primary coverts and tail tip [83]. Although *Orthopsittaca* is mostly green, its greater primary coverts are also bluish [83]. It seems that the blue or violet-blue colouration of greater primary coverts is a common feature of the clade including *Aratinga* and three macaws, and could be present in its common ancestor. *Eupsittula*, which is sister to this clade in the trees with 11 taxa, also has blue greater wing coverts, like *Aratinga*. However, the blue is paler, reduced to a faint tinge in cactorum and does not extend to the outer primary coverts [5].

**Fig. 10:**
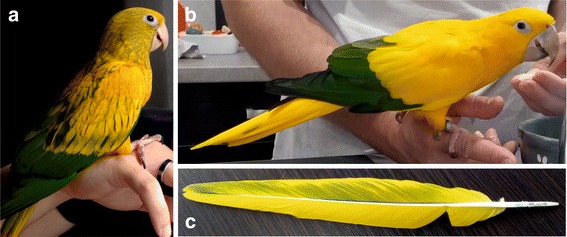
*Guaruba guarouba* colouration. **a**: Juvenile individual with mixed yellow and green plumage. **b** Adult with typical mainly yellow plumage**. c** Double coloured feather from an adult

In contrast to *Aratinga*, *Eupsittula* and three macaw genera, the greater primary coverts, as well as primaries and secondaries of *Psittacara*, *Thectocercus* and *Guaruba* are green [83]. However, other body parts, including the tail, are mainly yellow in adult *Guaruba* (Fig. 10b) unlike *Psittacara* and *Thectocercus*, which are predominantly green. *Thectocercus*’s tail is additionally red in its inner webs. Nevertheless, it is quite probable that *Guaruba*’s yellow colouration evolved from an ancestral green-coloured state because *Guaruba* chicks and juvenile individuals have mixed yellow and green plumage (Fig. 10a). Moreover, some double-coloured feathers can be found even in individuals much older than juveniles (Fig. 10c). Therefore, it seems probable that the green colouration is an ancestral feature of the clade *Psittacara*-*Thectocercus*-*Guaruba*.

**Fig. 11:**
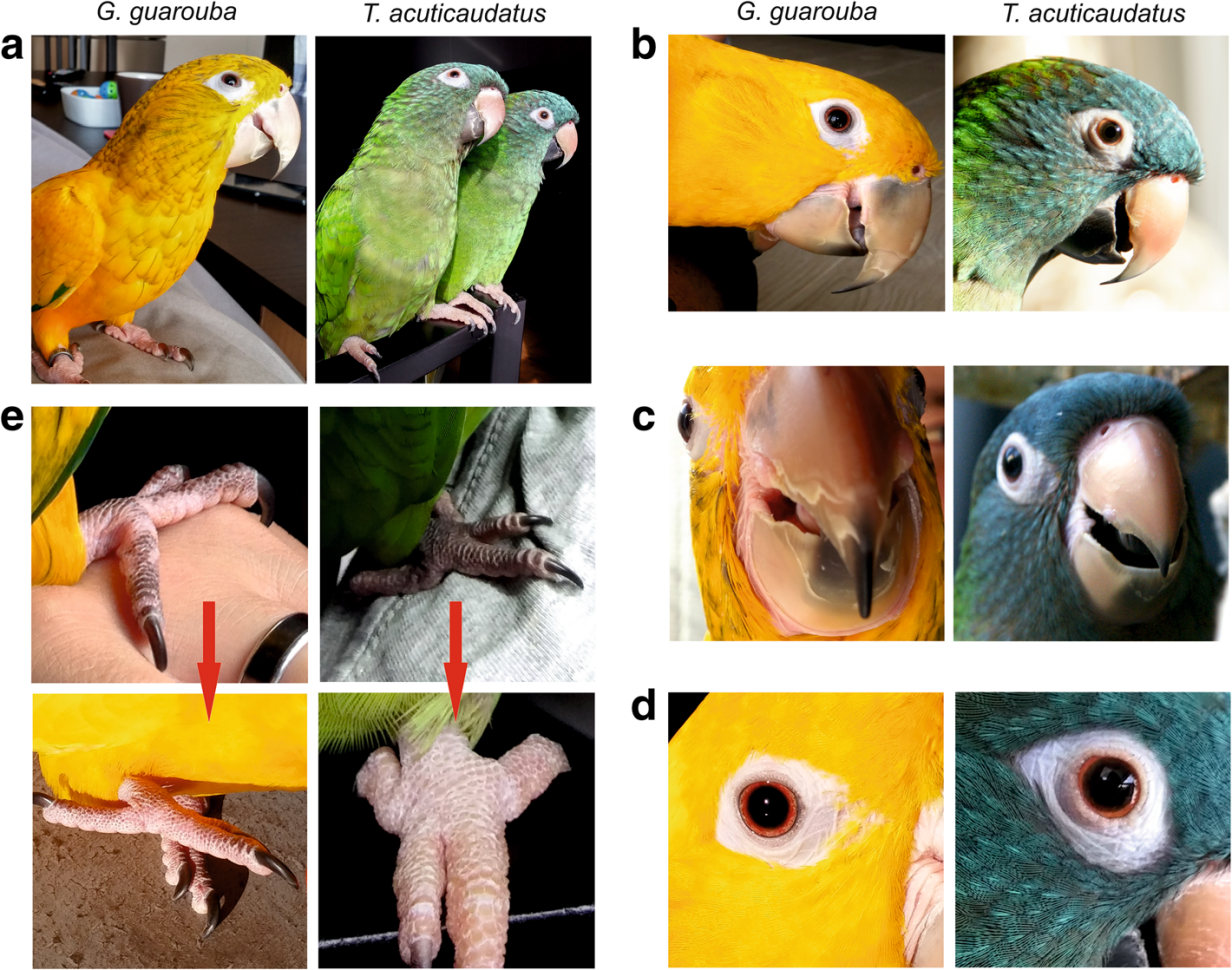
Morphological comparison of *Guaruba guarouba* and *Thectocercus acuticaudatus acuticaudatus*. **a** Body shape. **b** Head with bill from the lateral view. **c** Bill from the front view. **d** Eye. **e** Change of leg colour from grey in juvenile to flesh-pink in adult

**Fig. 12:**
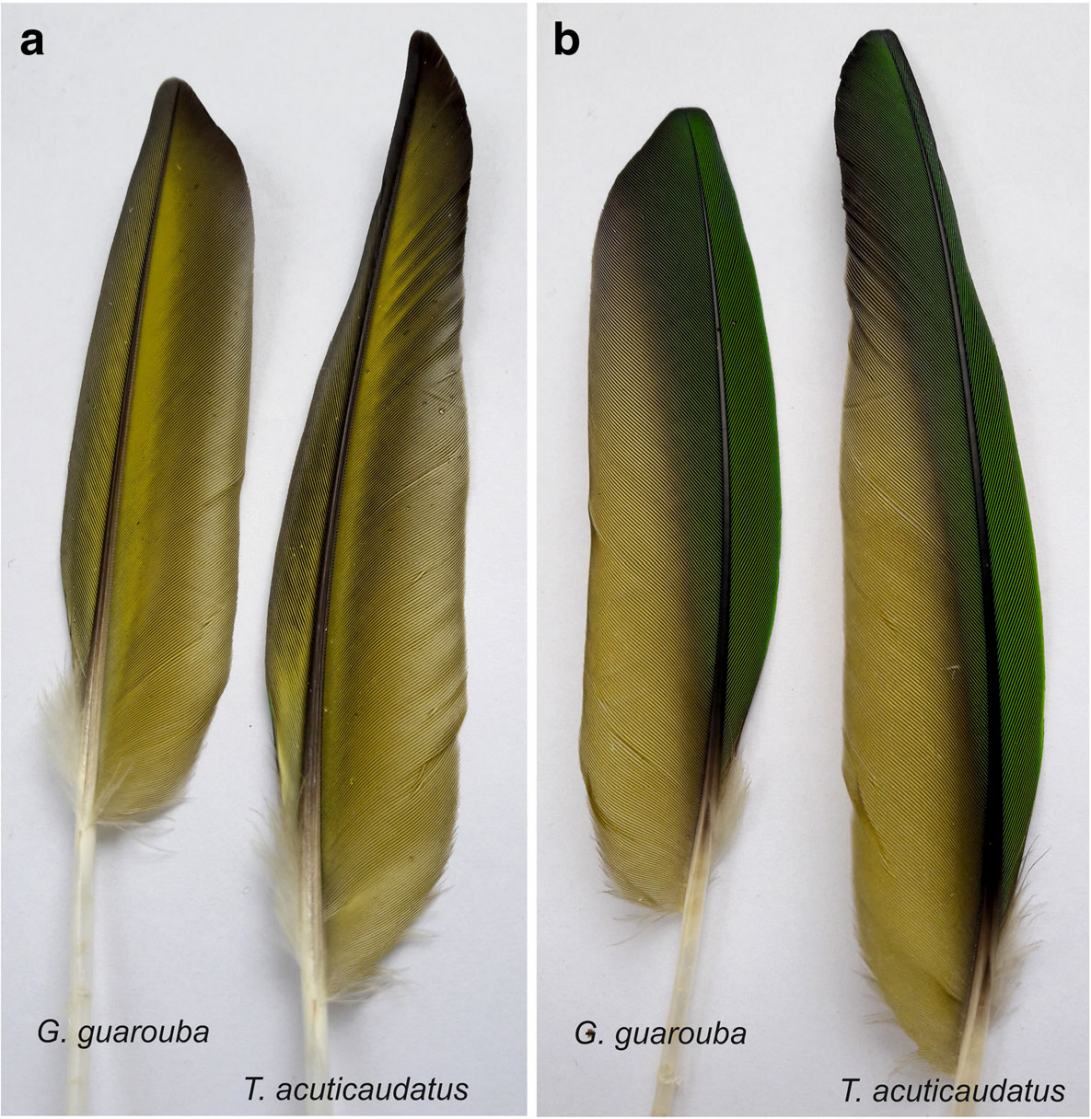
Colouration of remiges in *Guaruba guarouba*-underside (**a**) and upperside (**b**)

*Guaruba* and *Thectocercus*, which are closely related in phylogenetic trees, share many similar morphological features, such as: relatively large size compared to other members of previously broadly defined *Aratinga*; fusiform body shape with strongly build chest (Fig. 11a); bill shape from the lateral view (Fig. 11b); very wide bill, whose lower mandible is more sizeable in width than in depth (Fig. 11c); slender, acute and ridged tip of the upper mandible (Fig. 11b); large and whitish eye ring (Fig. 11d); orange eye iris (Fig. 11d); underside (Fig. 12a) and upperside (Fig. 12b) colouration of remiges. These genera are characterized also by some interesting developmental features. A grey colour of the legs in young individuals is replaced by a flesh-pink colour in mature individuals (Fig. 11e). *Guaruba*’s upper mandible horn-coloured with grey-coloured tip (Fig. 11b, c), resembles that of all the five recognized *Thectocercus* subspecies. The lower mandible of *Guaruba* is generally also horn-coloured, but a small grey area close to the edge of its central part is often noticeable (Fig. 11b, c). Only two *Thectocercus* subspecies share this horn-grey pattern of the lower mandible, whereas three other subspecies have the lower mandible dark grey (Fig. 11a, b). However, their young chicks also display the horn-coloured state of the lower mandible [83], which is subsequently replaced by the grey colouration with the intermediate horn-grey pattern observed in *Guaruba* (Fig. 11b, c).

Since the basal taxa *Pyrrhura* and *Rhynchopsitta* have mainly a green plumage, an ancestral state of the *Arini* was most probably of green colouration. This feature survived in related *Psittacara* and *Thectocercus*; in *Guaruba*, *Eupsittula* and the lineage *Aratinga* + macaws, however, more differentiated colouring evolved, including blue, violet, yellow, black, brown and red.

## Conclusions

Using the complete mitochondrial genomes of the main representatives of the tribe *Arini*, we validated the usefulness and applicability of different phylogenetic methods and various molecular markers in the phylogeny of parrots. The individual markers provided conflicting tree topologies, and different tree-building methods produced various topologies even for the same marker. Therefore, the results of phylogenetic analyses relying on single approaches and markers need to be interpreted with caution. It applies in particular to mitochondrial *cytb*, *nd2* and *nd6* genes, commonly used in parrot phylogenies, because the tree topologies based on them showed a substantial disagreement across the methods used and a sharp difference from the tree based on all molecular markers. Among individual markers, the strongest phylogenetic signal was recorded in the control region as well as in tRNA and 16S rRNA genes, but the trees based on them were not fully resolved either. Uncertainty in the inferred phylogenies may arise from taxon sampling, rapid evolution of the taxa under study and a short time interval between the divergences of particular lineages. The best resolved phylogenetic relationships of these parrots can be obtained only on the concatenated set of all mitochondrial markers, especially with the application of robust methods such as Bayesian and maximum likelihood. The molecular phylogenies are supported by several features in the mitochondrial genomes and are consistent with plumage colouration, which may reflect an agreement between the mtDNA phylogenies and the species tree. However, it seems advisable to incorporate additional molecular markers to verify the phylogenetic relationships within *Arini* presented in this paper.

## Additional files

Additional file 1: Comparison of mitochondrial genomes from ten *Arini* representatives. (PDF 5796 kb)

Additional file 2: Sequence data sets and nucleotide substitution models used in the present study. (PDF 53 kb)

Additional file 3: Tree topologies obtained by eight methods for individual mitochondrial markers from 7 parrot taxa. When some methods yielded several equally viable topologies, the majority-rule consensus tree was presented. The following phylogenetic approaches were applied: Bayesian analyses in MrBayes (MB) and PhyloBayes (PB), maximum likelihood analyses with partitioned data in TreeFinder (TF) and not partitioned in PAUP (ML) as well as neighbour joining (NJ), minimum evolution (ME), weighted least squares (WLS) and maximum parsimony (MP) in PAUP. The same tree topologies have the same background colour. (PDF 32 kb)

Additional file 4: Tree topologies built employing eight methods for individual mitochondrial markers from 11 parrot taxa. When some methods yielded several equally probable topologies, the majority-rule consensus tree was presented. The following phylogenetic approaches were applied: Bayesian analyses in MrBayes (MB) and PhyloBayes (PB), maximum likelihood analyses with partitioned data in TreeFinder (TF) and not partitioned in PAUP (ML) as well as neighbour joining (NJ), minimum evolution (ME), weighted least squares (WLS) and maximum parsimony (MP) in PAUP. The same tree topologies have the same background colour. (PDF 647 kb)

Additional file 5: Data set with 7-taxa: distance of individual marker trees from trees A and B based on the concatenated alignment of all markers. The maximum distance = 8. When some methods yielded several equally probable topologies, we averaged the calculated distances for these trees. The following phylogenetic approaches were applied: Bayesian analyses in MrBayes (MB) and PhyloBayes (PB), maximum likelihood analyses with partitioned data in TreeFinder (TF) and not partitioned in PAUP (ML) as well as neighbour joining (NJ), minimum evolution (ME), weighted least squares (WLS) and maximum parsimony (MP) in PAUP. (PDF 49 kb)

Additional file 6: Data set with 11 taxa: distance of individual marker trees from trees F, G and H based on the concatenated alignment of all markers. The maximum distance = 16. When some methods yielded several equally probable topologies, we averaged the calculated distances for these trees. The following phylogenetic approaches were applied: Bayesian analyses in MrBayes (MB) and PhyloBayes (PB), maximum likelihood analyses with partitioned data in TreeFinder (TF) and not partitioned in PAUP (ML) as well as neighbour joining (NJ), minimum evolution (ME), weighted least squares (WLS) and maximum parsimony (MP) in PAUP. (PDF 53 kb)

## Abbreviations

ALL set: the concatenated set of all nucleotide markers
ALL-CR: the set of all markers excluding the control region
BP: bootstrap percentage
CA: correspondence analysis
CR: control region
ME: minimum evolution
MP: maximum parsimony
NJ: neighbour joining
PCGs: protein-coding genes
PP: posterior probability
WLS: weighted least squares
