# Supplementary material for: The influence of molecular markers and methods on inferring the phylogenetic relationships between the representatives of the Arini (parrots, Psittaciformes), determined on the basis of their complete mitochondrial genomes
Source: BMC Evol Biol. 2017 Jul 14;17:166. doi: 10.1186/s12862-017-1012-1 (PMC5513162; doi:10.1186/s12862-017-1012-1)
Supplement: Supplementary file 1 — Comparison of mitochondrial genomes from ten Arini representatives. (PDF 5796 kb) [file 12862_2017_1012_MOESM1_ESM.pdf]

Comparison of mitochondrial genomes from ten *Arini* representatives

1. General organization of *Arini* mitochondrial genomes

The studied *Arini* genomes have comparable length (Table 1). The largest difference is 47 nucleotides. All ten mitogenomes contain the same genetic elements: 13 protein-coding genes (PCGs), 22 tRNA genes, 2 rRNA genes and the control region. These elements show the same order and orientation. The sense strands of genes for two rRNAs, ND6 protein and eight tRNAs are located on the heavy strand (richer in guanine), whereas the other 12 protein-coding genes and 14 tRNA genes are encoded on the light strand (poorer in guanine).

The overall base composition of the compared *Arini* genomes is very similar but not identical. *Rhynchopsitta terrisi* genome is characterized by the highest A+T content, whereas *Pyrrhura rupicola* by the lowest (Table 1). The maximal difference between the genomes is 2.4%. Much greater difference in A+T content is demonstrated by individual genes (Figure 1). The largest difference between the maximum and minimum of A+T% values was found for tRNA-Ser1 and tRNA-Glu genes: 9.0% and 8.6%, respectively. Among protein-coding genes, *atp6* (5.3%) and *nd3* (4.6%) showed the greatest difference. The lowest A+T% variation is in 16S rRNA (1.1%), and among PCGs, in *nd5* (2.1%) and *nd4* (2.2%). The asymmetry of DNA strand measured by normalized differences between complementary nucleotides, i.e. GC skew and AT skew is very similar in the *Arini* genomes (Table 1). GC skew ranges from -0.37 to -0.41, and AT skew from 0.09 to 0.16. *Pyrrhura rupicola* demonstrates the greatest skews and *Rhynchopsitta terrisi* the smallest.

Among the compared species, the genome from *Rhynchopsitta terrisi* is the most diverged. Its percent distance to others is more than 11.5% (Table 2). The smallest distance (9.7%) is shown by the pairs *Guaruba guarouba* - *Thectocercus acuticaudatus* and *Ara glaucogularis* - *Primolius couloni*.

2. Sequence features of protein-coding genes

The usage of initial and termination codons and lengths of protein-coding genes (PCGs) are presented in Table 3. Eleven of them use the same start codons in all ten mitogenomes. Only *Eupsittula pertinax* and *Primolius couloni nd2* gene begins with ATG instead of ATA as in others and *Rhynchopsitta terrisi cox1* gene begins with ATG instead of commonly used GTG. ATG is the most common start codon found in nine PCGs. The *nd3* gene requires ATA and the *nd5* gene utilizes GTG as the start translation codon.

Stop codon usage in all ten *Arini* species is consistent in eight PCGs (*nd1*, *cox1*, *cox2*, *cox3*, *nd3*, *nd4*, *cytb* and *atp6*). The genes *cox2* and *cytb* use TAA codon, *nd1* and *cox1* end with AGG, and four other genes use the truncated TA_ (*atp6* and *nd3*) or T__ (*cox3* and *nd4*) stop codons. *Eupsittula pertinax* mitogenome stands out from the other nine parrots in respect of stop codons in *atp8* and *nd4L*. Both genes of this species end with truncated TA_ codons instead of the complete TAA codon, which is present in the genes from the other investigated *Arini* species.

Interestingly, gene *nd2* only from *Primolius couloni* has TAA codon instead of the truncated TA_ stop codon identified in all other analysed *Arini* mitogenomes. Similarly, gene *nd6* only from *Orthopsittaca manilata* utilizes TAA stop codon instead of TAG found in other species. Moreover, gene *nd5* from *Ara glaucogularis*, *Aratinga solstitialis* and *Orthopsittaca manilata* has TAG codon, whereas in other mitogenomes this gene ends with TAA.

Genes *nd3* in reptiles and birds usually do not have a translated adenine inside their sequence [1, 2]. We also detected such extra nucleotide in the *Arini* mitogenomes. However, this nucleotide is thymine in the case of *nd3* gene from *Eupsittula pertinax* and cytosine in other investigated species, i.e.: *Ara glaucogularis*, *Aratinga solstitialis*, *Guaruba guarouba*, *Orthopsittaca manilata*, *Primolius couloni*, *Psittacara mitratus*, *Pyrrhura rupicola*, *Rhynchopsitta terrisi* and *Thectocercus acuticaudatus*.

3. Intergenic regions and overlaps

Among 20 intergenic elements, the control region (CR) is the longest (Table 4). In all studied mitogenomes, it is located between tRNA-Glu and tRNA-Phe genes. *Psittacara mitratus* has the shortest CR and *Rhynchopsitta terrisi* the longest. The difference between them is 64 bp. Disregarding CR, the longest spacer with the length of 19 bp is present between *nd5* and *cytb* genes in *Psittacara mitratus* and the longest overlap (-10 bp) is in *atp8* and *atp6* genes. Among all identified intergenic elements, the overlap (-1 bp) for *cytb* and tRNA-Thr genes was found only in *Primolius couloni*. The mitogenomes from *Ara glaucogularis* and *Primolius couloni* share the spacer region between tRNA-Val and 16S rRNA genes. The length of another eight intergenic spacers and two overlaps are conserved across all studied mtDNAs. Seven other spacer regions vary in size in at least one of the ten mitogenomes: tRNA-Leu/ND1, tRNA-Ile/tRNA-Gln, tRNA-Ala/tRNA-Asn, tRNA-Asn/tRNA-Cys, tRNA-Tyr/*cox1*, tRNA-Ser/tRNA-Asp and *nd5*/*cytb*. The overlap between *atp8* and *atp6* genes has a variable length, too.

4. Transfer RNA and rRNA genes

Typical 22 tRNAs are present in the studied *Arini* mtDNA genomes including two different copies of tRNA-Leu and tRNA-Ser genes (Table 5). The length of tRNA genes varies from 66 bp (tRNA-Ser) to 75 bp (one of two tRNA-Leu copies). Only in the case of 14 tRNAs their length is consistent for all ten investigated *Arini* species. *Pyrrhura rupicola* mitogenome stands out from the others in respect of the length of tRNA-Asn and tRNA-Ser genes. *Primolius couloni* mitogenome differs from the others in the length of tRNA-Ser and tRNA-Thr genes. The *Psittacara mitratus* tRNA-Leu gene is one nucleotide longer than in the other nine species. The length of four other tRNAs (tRNA-Phe, tRNA-Val, tRNA-Lys and tRNA-Glu) differs in 3–4 nucleotides among the mitogenomes. Two rRNAs are also variable in length in the parrot species: 12S rRNA (up to 8 bp) and 16S rRNA (up to 10 bp).

5. Control region

The control region of *Arini* species can be divided into three domains with typical conserved sequence motifs, which were described in reference sequences [3-7]. The tRNA-Glu-adjacent domain I contains an extended terminal-associated sequence (ETAS), which is related to the termination of H-strand replication (Figure 2). Interestingly, the CSB-1-like motif, which is considered to be unique for bird domain I [8], was not found in the studied *Arini*. Although B-box, D-box and F-box are believed to be prevalent in birds, B-box was not identified within the analysed control regions. However, we found C-box and E-box, which were documented so far only in some birds [7]. Nevertheless, the C-box seems to be less conservative compared to other boxes and contains a deletion in eight species. Additionally, bird similarity box (BS-box) can be also identified in control regions from ten parrots. BS-box and D-box are the most conserved motifs in domain II. CSB-1 motif located at the 5′ end of domain III is also conserved in all ten examined *Arini* species. However, additional CSB motifs (CSB-2 and CSB-3), typically identified in mammals, were not found in our analyses. It is assumed that the short Conserved Sequence Blocks (CSB) regulate the initiation of replication and transcription of mitochondrial genome. Our results suggest that a dominant role in these processes is played by CSB-1 because it is well conserved in the *Arini* species.

Table 1. General characteristics of *Arini* mitogenomes compared in the study.

Species	Accession	Length [bp]	Nucleotide composition [%]							Reference
			T	C	A	G	A+T	GC skew[1]	AT skew[2]	
Ara glaucogularis	JQ782215	16,983	23.4	32.3	30.1	14.2	53.6	-0.39	0.13	[9]
Aratinga solstitialis	JX441869	16,984	23.8	31.9	30.3	14.0	54.1	-0.39	0.12	[10]
Eupsittula pertinax	HM640208	16,980	23.2	32.5	30.4	13.9	53.6	-0.40	0.13	[11]
Guaruba guarouba	JQ782217	17,008	23.4	32.2	30.4	14.0	53.8	-0.39	0.13	[12]
Orthopsittaca manilata	KJ579139	16,985	23.3	32.4	29.8	14.5	53.2	-0.38	0.12	[13]
Primolius couloni	KF836419	16,995	23.4	32.3	29.8	14.4	53.3	-0.38	0.12	[14]
Psittacara mitratus	JX215256	16,984	23.3	32.3	30.3	14.1	53.6	-0.39	0.13	[15]
Pyrrhura rupicola	KF751801	16,994	22.1	33.4	30.4	14.1	52.5	-0.41	0.16	[16]
Rhynchopsitta terrisi	KF010318	17,027	24.9	31.0	30.0	14.1	54.9	-0.37	0.09	[17]
Thectocercus acuticaudatus	JQ782214	16,998	23.4	32.3	30.3	14.0	53.7	-0.40	0.13	[18]

[1]GC skew = (G-C)/(G+C); [2]AT skew = (A-T)/(A+T)

Table 2. The percent of base differences between *Arini* mitogenomic sequences (below the diagonal) and their errors calculated by bootstrap method with 1000 replicates (above the diagonal) in MEGA [19].

**Species**	*A. g.*	*A. s.*	*E. p.*	*G. g.*	*O. m.*	*P. c.*	*P. m.*	*P. r.*	*R. t.*	*T. a.*
Ara glaucogularis		0.24	0.24	0.20	0.21	0.21	0.24	0.24	0.26	0.23
Aratinga solstitialis	9.60		0.22	0.17	0.18	0.18	0.21	0.21	0.21	0.16
Eupsittula pertinax	10.08	10.21		0.22	0.19	0.25	0.21	0.21	0.21	0.24
Guaruba guarouba	9.88	9.95	9.98		0.17	0.17	0.23	0.18	0.25	0.18
Orthopsittaca manilata	8.27	9.75	10.10	9.59		0.19	0.22	0.18	0.24	0.20
Primolius couloni	8.07	9.92	10.08	10.14	8.41		0.27	0.20	0.20	0.15
Psittacara mitratus	10.03	10.40	9.82	9.65	9.95	10.56		0.26	0.24	0.27
Pyrrhura rupicola	11.19	11.27	10.81	10.95	11.14	10.98	10.97		0.22	0.19
Rhynchopsitta terrisi	11.71	12.05	11.62	11.64	12.10	11.87	11.68	11.78		0.26
Thectocercus acuticaudatus	9.92	9.96	9.89	5.97	9.92	10.14	9.68	10.96	11.78	

Table 3. Length and start/stop codons in bp of the mitochondrial protein-coding genes from ten *Arini* representatives. Differences were indicated in **red**.

**Gene**	***Ara glaucogularis* *Aratinga solstitialis***		***Eupsittula pertinax***		***Guaruba guarouba* *Psittacara mitratus* *Pyrrhura rupicola* *Thectocercus acuticaudatus***		***Orthopsittaca manilata***		***Primolius couloni***		***Rhynchopsitta terrisi***	
	**Start**	**Stop**	**Start**	**Stop**	**Start**	**Stop**	**Start**	**Stop**	**Start**	**Stop**	**Start**	**Stop**
ATP6	ATG	TA_	ATG	TA_	ATG	TA_	ATG	TA_	ATG	TA_	ATG	TA_
ATP8	ATG	TAA	ATG	**TA_**	ATG	TAA	ATG	TAA	ATG	TAA	ATG	TAA
COX1	GTG	AGG	GTG	AGG	GTG	AGG	GTG	AGG	GTG	AGG	**ATG**	AGG
COX2	ATG	TAA	ATG	TAA	ATG	TAA	ATG	TAA	ATG	TAA	ATG	TAA
COX3	ATG	T__	ATG	T__	ATG	T__	ATG	T__	ATG	T__	ATG	T__
CYTB	ATG	TAA	ATG	TAA	ATG	TAA	ATG	TAA	ATG	TAA	ATG	TAA
ND1	ATG	AGG	ATG	AGG	ATG	AGG	ATG	AGG	ATG	AGG	ATG	AGG
ND2	ATA	TA_	**ATG**	TA_	ATA	TA_	ATA	TA_	**ATG**	**TAA**	ATA	TA_
ND3	ATA	TA_	ATA	TA_	ATA	TA_	ATA	TA_	ATA	TA_	ATA	TA_
ND4	ATG	T__	ATG	T__	ATG	T__	ATG	T__	ATG	T__	ATG	T__
ND4L	ATG	TAA	ATG	**TA_**	ATG	TAA	ATG	TAA	ATG	TAA	ATG	TAA
ND5	GTG	**TAG**	GTG	TAA	GTG	TAA	GTG	**TAG**	GTG	TAA	GTG	TAA
ND6	ATG	TAG	ATG	TAG	ATG	TAG	ATG	**TAA**	ATG	TAG	ATG	TAG

Table 4. Length of the control regions and intergenic elements (spacers and overlaps) in ten *Arini* mitogenomes. Differences were indicated in **red**.

Region	*Ara glaucogularis*	*Aratinga solstitialis*	*Eupsittula pertinax*	*Guaruba guarouba*	*Orthopsittaca manilata*	*Primolius couloni*	*Psittacara mitratus*	*Pyrrhura rupicola*	*Rhynchopsitta terrisi*	*Thectocercus acuticaudatus*
tRNA^{Phe}/12S rRNA	0	0	0	0	0	0	0	0	0	0
12S rRNA/tRNA^{Val}	0	0	0	0	0	0	0	0	0	0
tRNA^{Val} /16S rRNA	**1**	0	0	0	0	**1**	0	0	0	0
16S rRNA/tRNA^{Leu}	0	0	0	0	0	0	0	0	0	0
tRNA^{Leu}/ND1	6	6	6	6	**5**	6	6	6	6	6
ND1/tRNA^{Ile}	-2	-2	-2	-2	-2	-2	-2	-2	-2	-2
tRNA^{Ile}/tRNA^{Gln}	5	5	5	5	**3**	5	5	5	5	5
tRNA^{Gln}/tRNA^{Met}	0	0	0	0	0	0	0	0	0	0
tRNA^{Met}/ND2	0	0	0	0	0	0	0	0	0	0
ND2/tRNA^{Trp}	0	0	0	0	0	0	0	0	0	0
tRNA^{Trp}/tRNA^{Ala}	1	1	1	1	1	1	1	1	1	1
tRNA^{Ala}/tRNA^{Asn}	1	1	1	**2**	1	1	1	1	1	**2**
tRNA^{Asn}/tRNA^{Cys}	2	2	2	2	**1**	2	2	2	2	2
tRNA^{Cys}/tRNA^{Tyr}	0	0	0	0	0	0	0	0	0	0
tRNA^{Tyr}/COX1	9	**10**	9	9	9	9	9	9	9	9
COX1/tRNA^{Ser}	0	0	0	0	0	0	0	0	0	0
tRNA^{Ser}/tRNA^{Asp}	**3**	4	4	4	4	4	4	**3**	**3**	**3**
tRNA^{Asp}/COX2	2	2	2	2	2	2	2	2	2	2
COX2/tRNA^{Lys}	1	1	1	1	1	1	1	1	1	1
tRNA^{Lys}/ATP8	1	1	1	1	1	1	1	1	1	1
ATP8/ATP6	-10	-10	**-9**	-10	-10	-10	-10	-10	-10	-10
ATP6/COX3	0	0	0	0	0	0	0	0	0	0
COX3/tRNA^{Gly}	0	0	0	0	0	0	0	0	0	0
tRNA^{Gly}/ND3	0	0	0	0	0	0	0	0	0	0
ND3/tRNA^{Arg}	0	0	0	0	0	0	0	0	0	0
tRNA^{Arg}/ND4L	1	1	1	1	1	1	1	1	1	1
ND4L/ND4	-7	-7	-7	-7	-7	-7	-7	-7	-7	-7
ND4/tRNA^{His}	0	0	0	0	0	0	0	0	0	0
tRNA^{His}/tRNA^{Ser}	0	0	0	0	0	0	0	0	0	0
tRNA^{Ser}/tRNA^{Leu}	0	0	0	0	0	0	0	0	0	0
tRNA^{Leu}/ND5	0	0	0	0	0	0	0	0	0	0
ND5/CYTB	11	**10**	11	11	11	11	**19**	**13**	11	11
CYTB/tRNA^{Thr}	0	0	0	0	0	**-1**	0	0	0	0
tRNA^{Thr}/tRNA^{Pro}	4	4	4	4	4	4	4	4	4	4
tRNA^{Pro}/ND6	3	3	3	3	3	3	3	3	3	3
ND6/tRNA^{Glu}	1	1	1	1	1	1	1	1	1	1
tRNA^{Glu}/CR	0	0	0	0	0	0	0	0	0	0
CR	**1485**	**1482**	**1473**	**1493**	**1488**	**1490**	**1458**	**1492**	**1522**	**1482**
CR/tRNA^{Phe}	0	0	0	0	0	0	0	0	0	0

Table 5. Positions and the lengths (in nt) of RNAs identified in mitogenomes of ten *Arini* species. Genes with different length were shaded.

Gene	*Ara glaucogularis*	*Aratinga solstitialis*	*Eupsittula pertinax*	*Guaruba guarouba*	*Orthopsittaca manilata*	*Primolius couloni*	*Psittacara mitratus*	*Pyrrhura rupicola*	*Rhynchopsitta terrisi*	*Thectocercus acuticaudatus*
$tRNA^{Phe}$	66	67	70	67	70	66	67	66	67	67
12S rRNA	972	975	971	978	971	974	976	971	969	977
$tRNA^{Val}$	71	71	70	72	70	71	71	72	72	73
16S rRNA	1566	1565	1572	1572	1567	1569	1575	1567	1575	1575
$tRNA^{Leu}$	75	75	75	75	75	75	75	75	75	75
$tRNA^{Ile}$	72	72	72	72	72	72	72	72	72	72
$tRNA^{Gln}$	71	71	71	71	71	71	71	71	71	71
$tRNA^{Met}$	68	68	68	68	68	68	68	68	68	68
$tRNA^{Trp}$	71	71	71	71	71	71	71	71	71	71
$tRNA^{Ala}$	69	69	69	69	69	69	69	69	69	69
$tRNA^{Asn}$	74	74	74	74	74	74	74	75	74	74
$tRNA^{Cys}$	67	67	67	67	67	67	67	67	67	67
$tRNA^{Tyr}$	70	70	70	70	70	70	70	70	70	70
$tRNA^{Ser}$	67	67	67	67	67	68	67	68	67	67
$tRNA^{Asp}$	69	69	69	69	69	69	69	69	69	69
$tRNA^{Lys}$	69	69	70	70	69	68	71	69	69	70
$tRNA^{Gly}$	69	69	69	69	69	69	69	69	69	69
$tRNA^{Arg}$	70	70	70	70	70	70	70	70	70	70
$tRNA^{His}$	69	69	69	69	69	69	69	69	69	69
$tRNA^{Ser}$	66	66	66	66	66	66	66	66	66	66
$tRNA^{Leu}$	70	70	70	70	70	70	71	70	70	70
$tRNA^{Thr}$	68	68	68	68	68	69	68	68	68	68
$tRNA^{Pro}$	69	69	69	69	69	69	69	69	69	69
$tRNA^{Glu}$	70	71	70	70	70	70	73	70	70	70

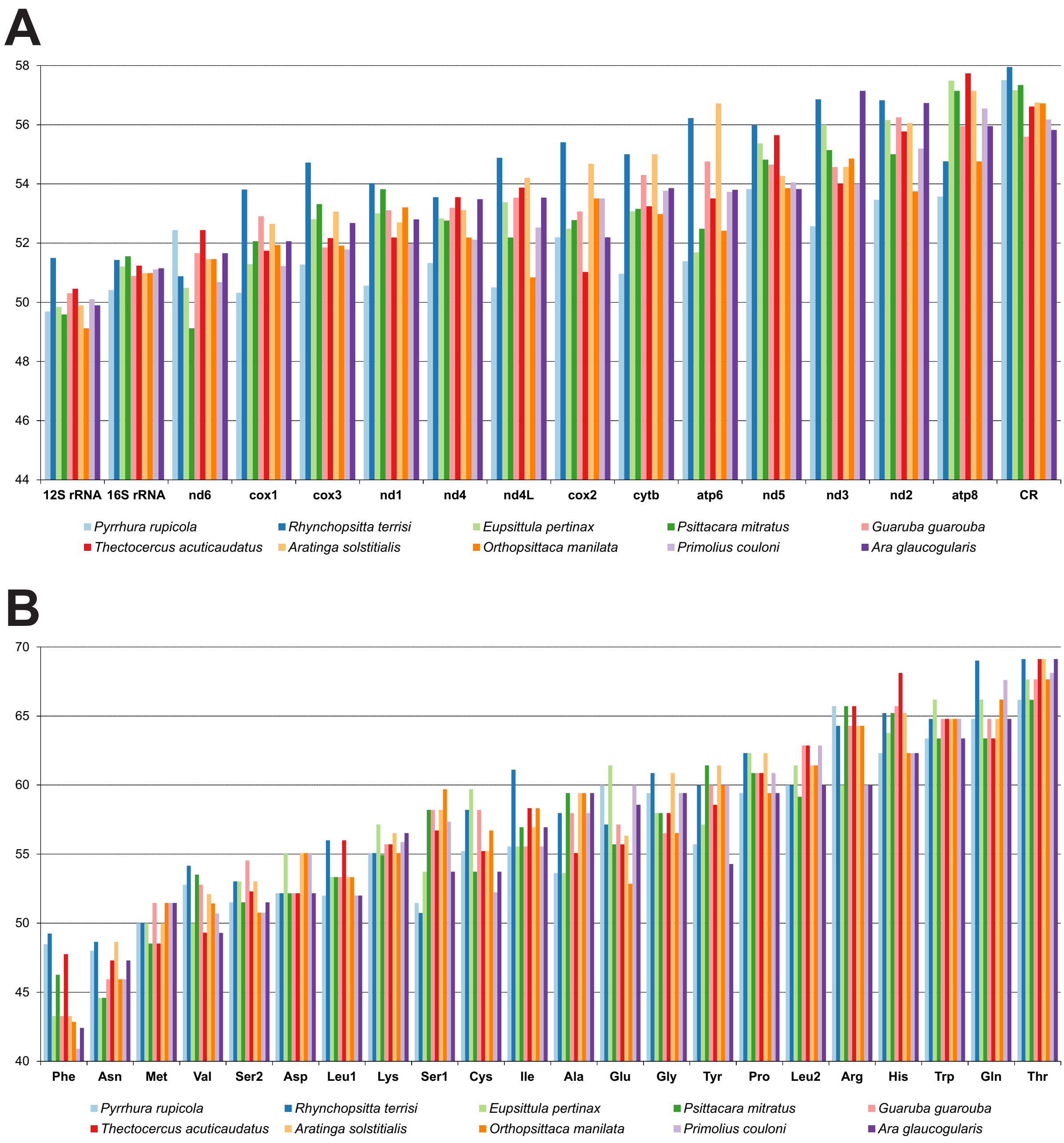

Figure 1. A+T% content of protein-coding genes, two rRNA genes and control region (A) as well as tRNA genes (B) in mitochondrial genomes of ten *Arini* representatives. The genes were arranged according to the average A+T%.

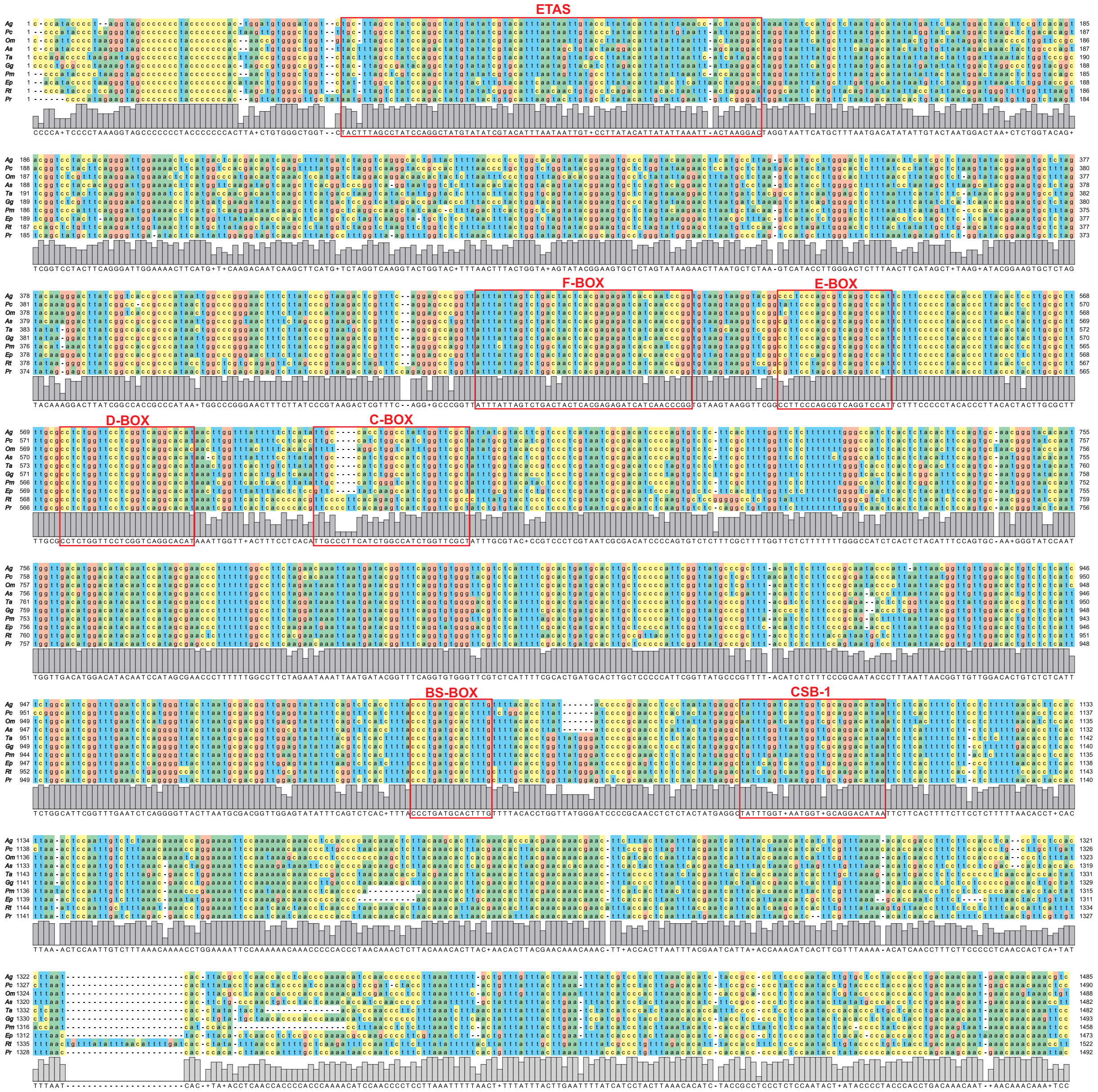

Figure 2. Multiple sequence alignment of mitochondrial control regions from ten Arini species: *Ara glaucogularis (Ag)*, *Primolius couloni (Pc)*, *Orthopsittaca manilata (Om)*, *Aratinga solstitialis (As)*, *Thectocercus acuticaudatus (Ta)*, *Guaruba guarouba (Gg)*, *Psittacara mitratus (Pm)*, *Eupsittula pertinax (Ep)*, *Rhynchopsitta terrisi (Rt)*, *Pyrrhura rupicola (Pr)*. Several conserved motifs were indicated: ETAS element as well as boxes F, E, D C, BS and CSB-1.

References

1. Mindell DP, Sorenson MD, Dimcheff DE: **An extra nucleotide is not translated in mitochondrial ND3 of some birds and turtles**. *Molecular Biology and Evolution* 1998, **15**(11):1568-1571.
2. Russell RD, Beckenbach AT: **Recoding of Translation in Turtle Mitochondrial Genomes: Programmed Frameshift Mutations and Evidence of a Modified Genetic Code**. *J Mol Evol* 2008, **67**(6):682-695.
3. Kan XZ, Li XF, Lei ZP, Wang M, Chen L, Gao H, Yang ZY: **Complete mitochondrial genome of Cabot's tragopan, Tragopan caboti (Galliformes: Phasianidae)**. *Genet Mol Res* 2010, **9**(2):1204-1216.
4. Roques S, Godoy JA, Negro JJ, Hiraldo F: **Organization and variation of the mitochondrial control region in two vulture species, Gypaetus barbatus and Neophron percnopterus**. *J Hered* 2004, **95**(4):332-337.
5. Yang R, Wu X, Yan P, Su X, Yang B: **Complete mitochondrial genome of Otis tarda (Gruiformes: Otididae) and phylogeny of Gruiformes inferred from mitochondrial DNA sequences**. *Molecular biology reports* 2010, **37**(7):3057-3066.
6. Ruokonen M, Kvist L: **Structure and evolution of the avian mitochondrial control region**. *Molecular phylogenetics and evolution* 2002, **23**:422-432.
7. Xiao B, Ma F, Sun Y, Li Q-W: **Comparative Analysis of Complete Mitochondrial DNA Control Region of Four Species of Strigiformes**. *Acta Genetica Sinica* 2006, **33**(11):965-974.
8. Delport W, Ferguson JWH, Bloomer P: **Characterization and evolution of the mitochondrial DNA control region in hornbills (Bucerotiformes)**. *J Mol Evol* 2002, **54**(6):794-806.
9. Urantowka AD: **Complete mitochondrial genome of Critically Endangered Blue- throated Macaw (Ara glaucogularis): its comparison with partial mitogenome of Scarlet Macaw (Ara macao).** *Mitochondrial DNA* 2016, **27**(1):422-424.
10. Urantowka AD, Strzala T, Kroczak A, Mackiewicz P: **The first complete genome of "true" Aratinga genus in comparison to mitogenomes of other parrots from Arini tribe.** *Mitochondrial DNA Part B: Resources* 2016, **1**(1):853–855.
11. Pacheco MA, Battistuzzi FU, Lentino M, Aguilar RF, Kumar S, Escalante AA: **Evolution of Modern Birds Revealed by Mitogenomics: Timing the Radiation and Origin of Major Orders**. *Molecular biology and evolution* 2011, **28**(6):1927-1942.
12. Urantowka AD, Strzala T, Mackiewicz P: **Complete mitochondrial genome of golden conure (Guaruba guarouba).** *Mitochondrial DNA Part B: Resources* 2017, **2**(1):33-34.
13. Urantowka AD: **Complete mitochondrial genome of Red-bellied Macaw (Orthopsittaca manilata): its comparison with mitogenome of Blue-throated Macaw (Ara glaucogularis)**. *Mitochondrial DNA A* 2016, **27**(3):2110-2111.
14. Urantowka AD: **Complete mitochondrial genome of Blue-headed Macaw (Primolius couloni): its comparison with mitogenome of Blue-throated Macaw (Ara glaucogularis)**. *Mitochondrial DNA A* 2016, **27**(3):2106-2107.
15. Urantowka AD, Mackiewicz P, Strzala T: **Complete mitochondrial genome of Mitred Conure (Psittacara mitratus): its comparison with mitogenome of Socorro Conure (Psittacara brevipes).** *Mitochondrial DNA A DNA MappSeq Anal* 2016, **27**(5):3363-3364.
16. Urantowka AD, Strzala T, Grabowski KA: **The first complete mitochondrial genome of Pyrrhura sp. - question about conspecificity in the light of hybridization between Pyrrhura molinae and Pyrrhura rupicola species**. *Mitochondrial DNA* 2016, **27**(1):471-473.
17. Urantowka AD, Strzala T, Grabowski KA: **Complete mitochondrial genome of endangered Maroon-fronted Parrot (Rhynchopsitta terrisi) - conspecific relation of the species with Thick-billed Parrot (Rhynchopsitta pachyrhyncha)**. *Mitochondrial DNA* 2014, **25**(6):424-426.
18. Urantowka AD, Grabowski KA, Strzala T: **Complete mitochondrial genome of Blue-crowned Parakeet (Aratinga acuticaudata)--phylogenetic position of the species among parrots group called Conures**. *Mitochondrial DNA* 2013, **24**(4):336-338.
19. Tamura K, Stecher G, Peterson D, Filipski A, Kumar S: **MEGA6: Molecular Evolutionary Genetics Analysis version 6.0**. *Molecular Biology and Evolution* 2013, **30**(12):2725-2729.
