# Supplementary material for: The influence of molecular markers and methods on inferring the phylogenetic relationships between the representatives of the Arini (parrots, Psittaciformes), determined on the basis of their complete mitochondrial genomes
Source: BMC Evol Biol. 2017 Jul 14;17:166. doi: 10.1186/s12862-017-1012-1 (PMC5513162; doi:10.1186/s12862-017-1012-1)
Supplement: Supplementary file 2 — Sequence data sets and nucleotide substitution models used in the present study. (PDF 53 kb) [file 12862_2017_1012_MOESM2_ESM.pdf]

Additional File 2. Sequence data sets used in the studies with applied substitution models in four programs.

Data set	Markers	Alignment length [bp]	Substitution models used in:			
			TreeFinder	PAUP	MrBayes[5]	PhyloBayes
7 taxa	*atp6*	684	J3+I,HKY+I,J3+I	010020+I	Mix+:I,I,I	CAT+GTR+Γ
	atp8	168	TN,HKY+I,HKY+I	010022+I	Mix+:E,I,I	CAT+GTR+Γ
	cox1	1,584	TN+I,HKY+I,GTR+I	012210+Γ	Mix+:I,E,I	CAT+GTR+Γ
	cox2	684	J2+I,TN+I,HKY+Γ	010020+I	Mix+:I,I,Γ	CAT+GTR+Γ
	cox3	784	J3+I,HKY,J1+I	010210+I	Mix+:Γ,E,I	CAT+GTR+Γ
	cytb	1,140	J3+Γ,J1+I,J1+Γ	010212+Γ	Mix+:I,I,I	CAT+GTR+Γ
	nd1	981	HKY+I,TN+Γ+I,HKY+Γ	HKY+Γ	Mix+:Γ,Γ,Γ	CAT+GTR+Γ
	nd2	1,040	J1+Γ,J1+Γ+I,J3+Γ	012034+Γ+I	Mix+:Γ,Γ,Γ	CAT+GTR+Γ
	nd3	350	HKY+I,HKY+I,J3+Γ	010020+I	Mix+:I,I,I	CAT+GTR+Γ
	nd4	1,393	J1+Γ,J1+Γ+I,TN+Γ+I	010020+I	Mix+:Γ,I,I	CAT+GTR+Γ
	nd4L	297	HKY,HKY+I,HKY	012212+Γ	Mix+:E,I,E	CAT+GTR+Γ
	nd5	1,815	J3+Γ,HKY+Γ+I,J1+Γ+I	010232+Γ+I	Mix+:Γ,Γ+I,Γ+I	CAT+GTR+Γ
	nd6	513	HKY+Γ,J3+I,J2+Γ	012340+Γ	Mix+:Γ,I,Γ	CAT+GTR+Γ
	12s rRNA	971	J3+Γ	010023+Γ+I	Mix+Γ+I	GTR+Γ
	16s rRNA	1,567	GTR+Γ	010234+Γ	Mix+Γ	GTR+Γ
	tRNA1[1]	560	HKY+Γ	HKY+Γ+I	Mix+Γ+I	GTR+Γ
	tRNA2[2]	983	J1+Γ	012232+Γ+I	Mix+Γ+I	GTR+Γ
	control region (CR)	1,478	TVM+Γ	010213+Γ+I	Mix+Γ+I	GTR+Γ
	ALL[3]	16,956	GTR+Γ+I,J1+Γ+I,J1+Γ+I,HKY+Γ,J3+I,J2+Γ,J3+Γ,GTR+Γ,TVM+Γ,HKY+Γ,J1+Γ	012310+Γ	Mix+:Γ+I,Γ+I,Γ+I,Γ,I,Γ,Γ+I,Γ,Γ+I,Γ+I,Γ+I	CAT+GTR+Γ
	ALL-CR[4]	15,478	GTR+Γ+I,J1+Γ+I,J1+Γ+I,HKY+Γ,J3+I,J2+Γ,J3+Γ,GTR+Γ,HKY+Γ,J1+Γ	012340+Γ	Mix+:Γ+I,Γ+I,Γ+I,Γ,I,Γ,Γ+I,Γ,Γ+I,Γ+I	CAT+GTR+Γ
11 taxa	*atp6*	684	HKY+Γ,J1+Γ+I,J3+Γ+I	010020+Γ+I	Mix+:Γ,I,Γ+I	CAT+GTR+Γ
	atp8	168	TN,HKY+I,HKY+I	010023+I	Mix+:E,I,I	CAT+GTR+Γ
	cox1	1,584	J3+Γ+I,HKY+I, TN+Γ+I	012310+Γ+I	Mix+:Γ+I,E,Γ	CAT+GTR+Γ
	cox2	684	J2+I,HKY+I,J3+Γ	HKY+Γ+I	Mix+:Γ,I,Γ	CAT+GTR+Γ
	cox3	784	J3+Γ,HKY+Γ+I,J1+Γ+I	010210+Γ+I	Mix+:Γ,I,Γ+I	CAT+GTR+Γ
	cytb	1,140	J2+Γ,J1+I,J1+Γ+I	010230+Γ+I	Mix+:I,I,I	CAT+GTR+Γ
	nd1	981	HKY+I,HKY+Γ+I,TVM+Γ+I	HKY+Γ+I	Mix+:I,I,Γ+I	CAT+GTR+Γ
	nd2	1,040	J1+Γ,J1+Γ+I,J3+Γ+I	010023+Γ+I	Mix+:Γ,Γ+I,Γ	CAT+GTR+Γ
	nd3	350	HKY+Γ,HKY+Γ,J1+Γ	010230+Γ+I	Mix+:Γ,Γ,Γ	CAT+GTR+Γ
	nd4	1,393	J1+Γ,J1+Γ+I,TN+Γ+I	010020+Γ+I	Mix+:Γ,Γ+I,Γ+I	CAT+GTR+Γ
	nd4L	297	HKY+Γ,HKY+I,HKY+I	012212+Γ	Mix+:E,E,I	CAT+GTR+Γ
	nd5	1,815	J2+Γ+I,HKY+Γ+I,J1+Γ+I	010212+Γ+I	Mix+:Γ,Γ+I,Γ+I	CAT+GTR+Γ
	nd6	513	J3+Γ,HKY+Γ,J2+Γ	010012+Γ+I	Mix+:Γ,Γ,Γ	CAT+GTR+Γ
	12s rRNA	962	GTR+Γ	010023+Γ	Mix+Γ	GTR+Γ
	16s rRNA	1,568	GTR+Γ	010234+Γ+I	Mix+Γ+I	GTR+Γ
	tRNA1[1]	560	HKY+Γ	012010+Γ+I	Mix+Γ+I	GTR+Γ
	tRNA2[2]	988	J1+Γ	012032+Γ+I	Mix+Γ+I	GTR+Γ
	control region (CR)	1,477	J2+Γ	010213+Γ	Mix+Γ	GTR+Γ
	ALL[3]	16,952	GTR+Γ+I,J1+Γ+I,GTR+Γ+I,J3+Γ,TVM+Γ,J2+Γ,J1+Γ,GTR+Γ,J2+Γ,HKY+Γ+I,J1+Γ	012314+Γ+I	Mix+:Γ+I,Γ+I,Γ+I,Γ,Γ,Γ,Γ,Γ+I,Γ,Γ+I,Γ+I	CAT+GTR+Γ
	ALL-CR[4]	15,475	GTR+Γ+I,J1+Γ+I,GTR+Γ+I,J3+Γ,TVM+Γ,J2+Γ,GTR+Γ,GTR+Γ,TVM+Γ+I,J1+Γ	012310+Γ+I	Mix+:Γ+I,Γ+I,Γ+I,Γ,Γ,Γ,Γ,Γ+I,Γ+I,Γ+I	CAT+GTR+Γ

[1] - includes tRNA-Ala, tRNA-Asn, tRNA-Cys, tRNA-Gln, tRNA-Glu, tRNA-Pro, tRNA-Ser1 and tRNA-Tyr; [2] - includes tRNA-Arg, tRNA-Asp, tRNA-Gly, tRNA-His, tRNA-Ile, tRNA-Leu1, tRNA-Leu2, tRNA-Lys, tRNA-Met, tRNA-Phe, tRNA-Ser2, tRNA-Thr, tRNA-Trp and tRNA-Val; [3] – includes all the nucleotide markers; [4] – the same as [3] but without CR; [5] – E means equal rates across sites.
