# Supplementary figures and images for: The influence of molecular markers and methods on inferring the phylogenetic relationships between the representatives of the Arini (parrots, Psittaciformes), determined on the basis of their complete mitochondrial genomes
Source: BMC Evol Biol. 2017 Jul 14;17:166. doi: 10.1186/s12862-017-1012-1 (PMC5513162; doi:10.1186/s12862-017-1012-1)

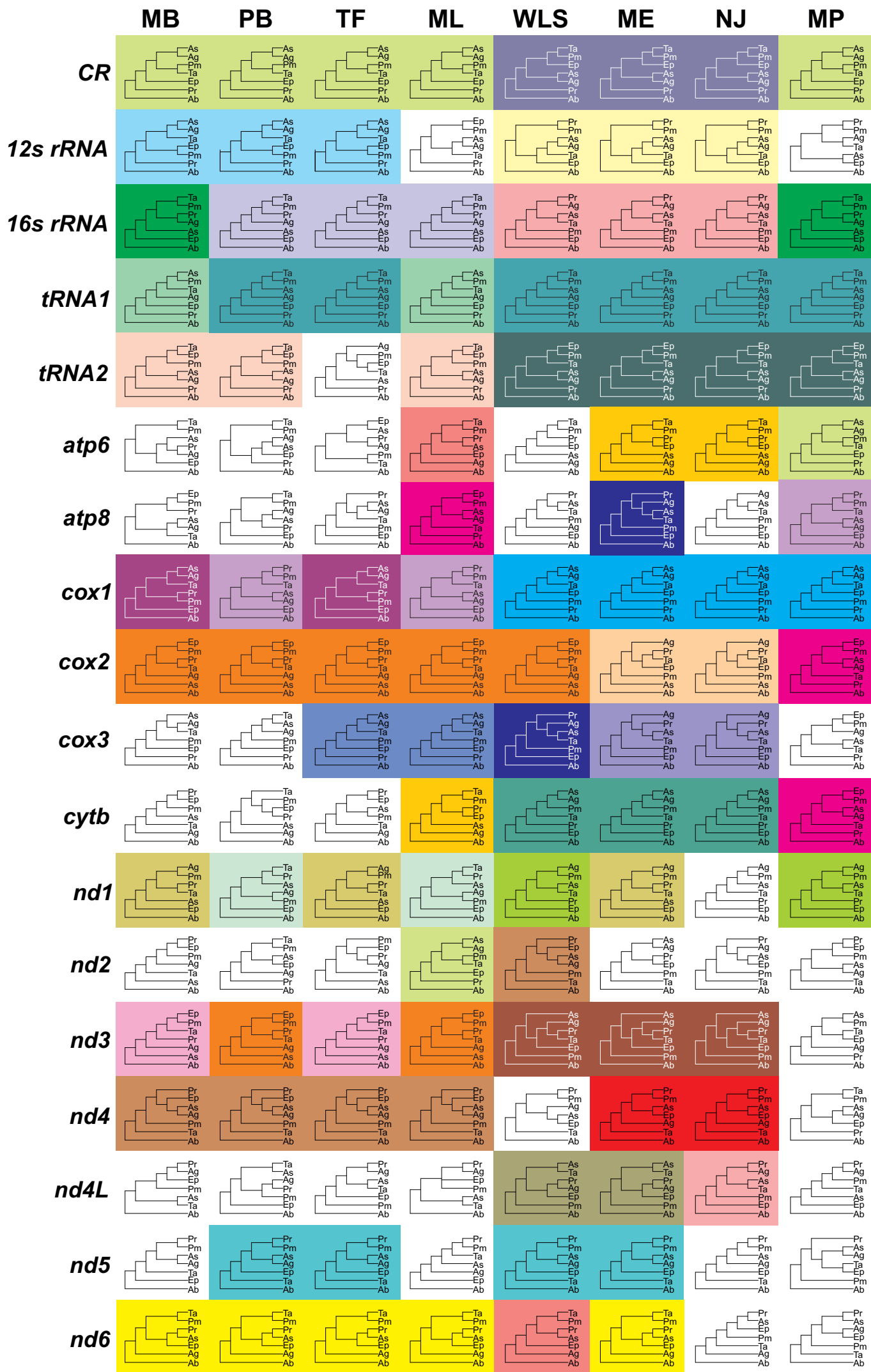

Supplement: Supplementary file 3 — Tree topologies obtained by eight methods for individual mitochondrial markers from 7 parrot taxa. When some methods yielded several equally viable topologies, the majority-rule consensus tree was presented. The following phylogenetic approaches were applied: Bayesian analyses in MrBayes (MB) and PhyloBayes (PB), maximum likelihood analyses with partitioned data in TreeFinder (TF) and not partitioned in PAUP (ML) as well as neighbour joining (NJ), minimum evolution (ME), weighted least squares (WLS) and maximum parsimony (MP) in PAUP. The same tree topologies have the same background colour. (PDF 32 kb) [file 12862_2017_1012_MOESM3_ESM.pdf]

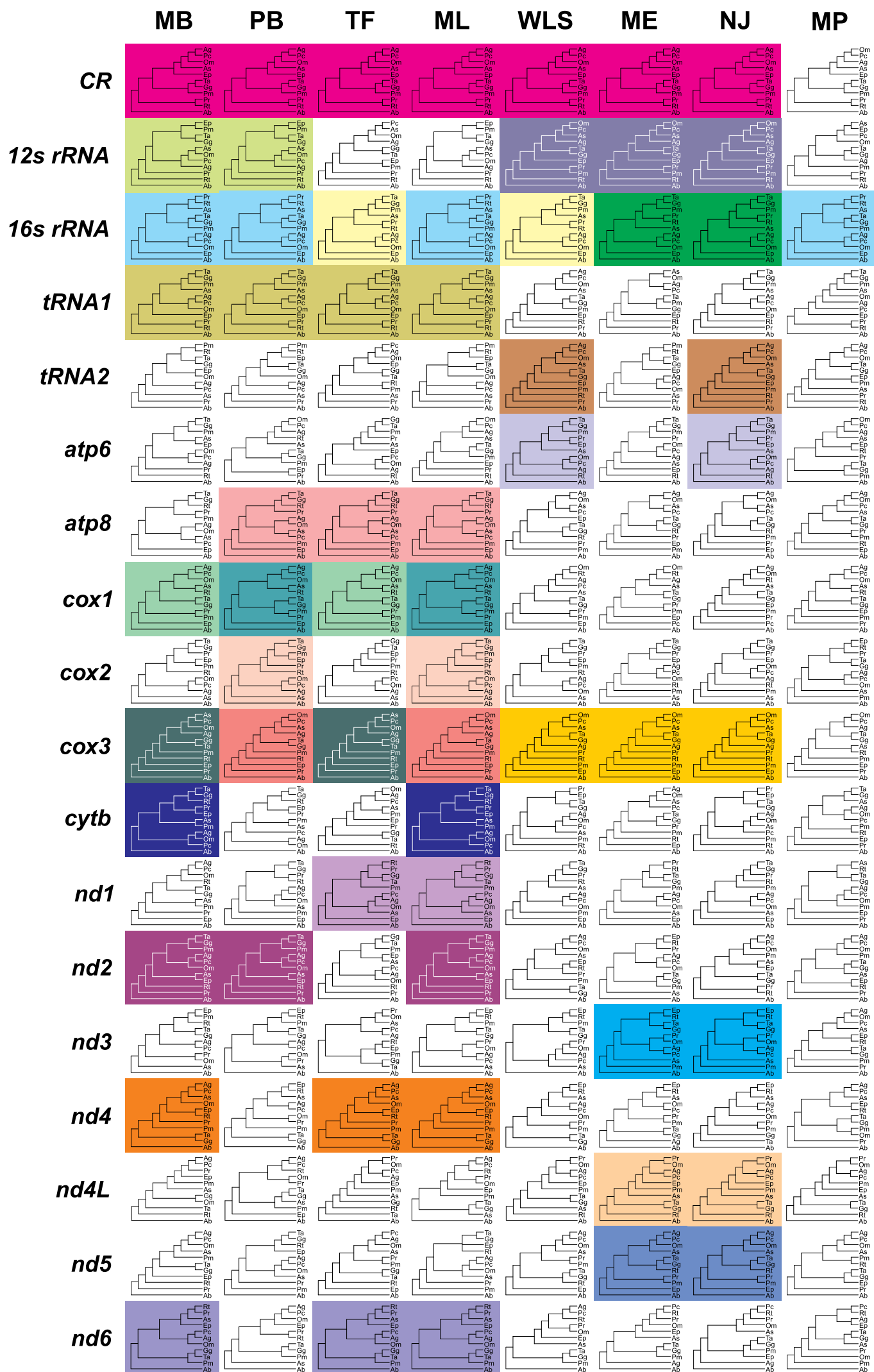

Supplement: Supplementary file 4 — Tree topologies built employing eight methods for individual mitochondrial markers from 11 parrot taxa. When some methods yielded several equally probable topologies, the majority-rule consensus tree was presented. The following phylogenetic approaches were applied: Bayesian analyses in MrBayes (MB) and PhyloBayes (PB), maximum likelihood analyses with partitioned data in TreeFinder (TF) and not partitioned in PAUP (ML) as well as neighbour joining (NJ), minimum evolution (ME), weighted least squares (WLS) and maximum parsimony (MP) in PAUP. The same tree topologies have the same background colour. (PDF 647 kb) [file 12862_2017_1012_MOESM4_ESM.pdf]
