# Supplementary material for: The influence of molecular markers and methods on inferring the phylogenetic relationships between the representatives of the Arini (parrots, Psittaciformes), determined on the basis of their complete mitochondrial genomes
Source: BMC Evol Biol. 2017 Jul 14;17:166. doi: 10.1186/s12862-017-1012-1 (PMC5513162; doi:10.1186/s12862-017-1012-1)
Supplement: Supplementary file 5 — Data set with 7-taxa: distance of individual marker trees from trees A and B based on the concatenated alignment of all markers. The maximum distance = 8. When some methods yielded several equally probable topologies, we averaged the calculated distances for these trees. The following phylogenetic approaches were applied: Bayesian analyses in MrBayes (MB) and PhyloBayes (PB), maximum likelihood analyses with partitioned data in TreeFinder (TF) and not partitioned in PAUP (ML) as well as neighbour joining (NJ), minimum evolution (ME), weighted least squares (WLS) and maximum parsimony (MP) in PAUP. (PDF 49 kb) [file 12862_2017_1012_MOESM5_ESM.pdf]

Additional file 5. Distances between trees with 7 taxa obtained by different methods for particular markers to trees (A and B) based on the concatenated alignment of all markers. The maximum distance = 8. When some methods proposed several equally probable topologies, we averaged the calculated distances for these trees. The eight phylogenetic approaches were applied: Bayesian analyses in MrBayes (MB) and PhyloBayes (PB), maximum likelihood analyses with partitioned data in TreeFinder (TF) and not partitioned in PAUP (ML) as well as neighbour joining (NJ), minimum evolution (ME), weighted least squares (WLS) and maximum parsimony (MP) in PAUP.

	Tree topology A								Tree topology B							
	MB	PB	TF	ML	WLS	ME	NJ	MP	MB	PB	TF	ML	WLS	ME	NJ	MP
CR	2	2	2	2	0	0	0	2	0	0	0	0	2	2	2	0
12s	4	4	4	4	6	6	6	7	4	4	4	4	6	6	6	7
16s	6	6	6	6	8	8	8	6	6	6	6	6	8	8	8	6
tRNA1	6	4	4	6	4	4	4	3.8	4	2	2	4	2	2	2	2.8
tRNA2	2	2	4	2	2	2	2	2	4	4	6	4	4	4	4	4
atp6	6	4	6	5.1	6	6	6	2	6	4	6	5.1	6	6	6	0
atp8	6	4	6	4	8	8	8	6.2	6	4	6	4	8	8	6	6
cox1	6	6	6	6	4	4	4	4.5	6	6	6	6	4	4	4	4
cox2	8	8	8	7	8	8	8	3.3	8	8	8	7.7	8	8	8	4
cox3	6	6	4	4	8	8	8	6	4	4	2	2	8	8	8	5.3
cytb	8	8	8	6	6	6	6	4	8	8	8	6	4	4	4	4
nd1	8	8	8	7.5	8	8	8	7	8	8	8	7.5	6	8	6	5
nd2	8	4	8	1.7	6	6	8	6	8	4	8	1	6	6	8	6
nd3	6	8	6	8	8	8	8	6	8	8	8	8	8	8	8	6
nd4	6	6	6	6	8	8	8	2	6	6	6	6	8	8	8	2
nd4L	8	8	8	7	8	8	8	7.7	8	8	8	7	8	8	8	7.7
nd5	6	6	6	8	6	6	8	6	6	6	6	8	6	6	8	6
nd6	6	6	6	6	6	6	8	8	6	6	6	6	6	6	8	8
