# Supplementary material for: The influence of molecular markers and methods on inferring the phylogenetic relationships between the representatives of the Arini (parrots, Psittaciformes), determined on the basis of their complete mitochondrial genomes
Source: BMC Evol Biol. 2017 Jul 14;17:166. doi: 10.1186/s12862-017-1012-1 (PMC5513162; doi:10.1186/s12862-017-1012-1)
Supplement: Supplementary file 6 — Data set with 11 taxa: distance of individual marker trees from trees F, G and H based on the concatenated alignment of all markers. The maximum distance = 16. When some methods yielded several equally probable topologies, we averaged the calculated distances for these trees. The following phylogenetic approaches were applied: Bayesian analyses in MrBayes (MB) and PhyloBayes (PB), maximum likelihood analyses with partitioned data in TreeFinder (TF) and not partitioned in PAUP (ML) as well as neighbour joining (NJ), minimum evolution (ME), weighted least squares (WLS) and maximum parsimony (MP) in PAUP. (PDF 53 kb) [file 12862_2017_1012_MOESM6_ESM.pdf]

Additional file 6. Distances between trees with 11 taxa obtained by different methods for particular markers to trees (F, G and H) based on the concatenated alignment of all markers. The maximum distance = 16. When some methods proposed several equally probable topologies, we averaged the calculated distances for these trees. The eight phylogenetic approaches were applied: Bayesian analyses in MrBayes (MB) and PhyloBayes (PB), maximum likelihood analyses with partitioned data in TreeFinder (TF) and not partitioned in PAUP (ML) as well as neighbour joining (NJ), minimum evolution (ME), weighted least squares (WLS) and maximum parsimony (MP) in PAUP.

	Tree topology F								Tree topology G								Tree topology H							
	MB	PB	TF	ML	WLS	ME	NJ	MP	MB	PB	TF	ML	WLS	ME	NJ	MP	MB	PB	TF	ML	WLS	ME	NJ	MP
CR	0	0	0	0	0	0	0	2	4	4	4	4	4	4	4	6	6	6	6	6	6	6	6	8
12s	10	10	10	10	12	12	12	10	10	10	10	10	12	12	12	12	8	8	6	8	10	10	10	12
16s	6	6	6	6	6	6	6	6.7	8	8	8	8	8	8	8	8.7	10	10	10	10	10	10	10	10.7
tRNA1	4	4	4	3.5	4	8	6	8	4	4	4	4.5	0	4	2	8	8	8	8	7.5	4	8	6	12
tRNA2	12	12	10	10	6	10	6	8.5	10	10	8	8	4	8	4	8.5	10	10	8	8	2	8	2	8
atp6	8	10	10	6	10	10	10	8	8	10	10	4	10	10	10	7	10	12	12	8	12	12	12	9
atp8	12	12	12	12	10	12	12	10.6	12	12	12	12	12	12	12	10.6	12	12	12	12	12	12	12	10.5
cox1	10	10	10	10	14	14	14	10	10	10	10	10	14	14	14	8	10	10	10	10	14	14	14	8
cox2	12	12	12	11.7	10	10	12	10	12	12	12	11.7	10	10	12	10	10	10	12	9.7	8	10	12	10
cox3	12	12	12	12	14	14	14	12	8	8	8	7.5	14	14	14	10	8	8	8	8.5	12	12	12	10
cytb	12	14	12	13	10	12	12	11	12	14	12	13	10	12	12	9	12	14	12	13	10	12	12	7
nd1	10	6	6	6	10	8	10	10	10	8	8	8	10	10	10	8	10	8	8	8	10	10	10	8
nd2	6	6	6	6	8	8	6	10	4	4	4	4	8	8	8	6	6	6	6	6	8	10	8	8
nd3	12	12	12	12	14	12	12	10	12	12	12	12	14	12	12	8.2	10	12	12	12	14	12	12	7.8
nd4	10	12	10	9.7	12	14	14	12	10	12	10	9.7	12	14	14	10	10	12	10	9.7	12	14	14	10
nd4L	14	12	14	10.7	12	12	12	12	14	12	14	10.7	12	12	12	12	14	12	14	10.7	12	12	12	12
nd5	6	8	8	7	8	8	8	8	2	8	8	7	8	8	8	6	4	8	8	7	8	8	8	6
nd6	6	6	6	5.5	10	12	12	14	8	8	8	7.5	10	12	12	14	10	10	10	9.5	12	14	14	14
